# Identifying crustal contributions in the Patagonian Chon Aike Silicic Large Igneous Province

**DOI:** 10.1007/s00410-023-02065-1

**Published:** 2023-10-24

**Authors:** Michelle L. Foley, Benita Putlitz, Lukas P. Baumgartner, Emiliano M. Renda, Alexey Ulianov, Guillaume Siron, Massimo Chiaradia

**Affiliations:** 1 Institute of Earth Sciences, University of Lausanne, UNIL-Mouline, 1015 Lausanne, Switzerland; 2 Department of Geosciences, University of Arizona, Tucson, AZ 85721 USA; 3 Instituto de Investigación en Paleobiología y Geología (UNRN-CONICET), Avenida Julio A. Roca 1242, R8332EXZ General Roca, Argentina; 4 WiscSIMS, Department of Geosciences, University of Wisconsin-Madison, Madison, WI 53706 USA; 5 Department of Biological, Geological, and Environmental Sciences, University of Bologna, 40126 Bologna, Italy; 6 Department of Earth Sciences, University of Geneva, Rue Des Maraîchers 13, 1205 Geneva, Switzerland

**Keywords:** Chon Aike Silicic Large Igneous Province, Zircon, Geochronology, Oxygen isotopes, Hafnium isotopes, Gondwana

## Abstract

The volcanic rocks of the Chon Aike Silicic Large Igneous Province (CASP) are recognized as magmas dominantly produced by crustal anatexis. Investigating the zircon of the CASP provides an opportunity to gain further insight into geochemical and isotopic differences of the potential magmatic sources (i.e., crust versus mantle), to identify crustal reservoirs that contributed to the felsic magmas during anatexis, and to quantify the contributions of the respective sources. We present a combined zircon oxygen and hafnium isotope and trace element dataset for 16 volcanic units of the two youngest volcanic phases in Patagonia, dated here with LA-ICP-MS U–Pb geochronology at ca. 148–153 Ma (El Quemado Complex, EQC) and ca. 159 Ma (western Chon Aike Formation, WCA). The EQC zircon have ^18^O-enriched values (δ^18^O from 7 to 9.5‰) with correspondingly negative initial εHf values (− 2.0 to − 8.0). The WCA zircon have δ^18^O values between 6 and 7‰ and εHf values ranging between − 4.0 and + 1.5. Binary δ^18^O-εHf mixing models require an average of 70 and 60% melt derived from partial melting of isotopically distinct metasedimentary basements for the EQC and WCA, respectively. Zircon trace element compositions are consistent with anatexis of sedimentary protoliths derived from LIL-depleted upper continental crustal sources. The overlap between a high heat flux environment (i.e., widespread extension and lithospheric thinning) during supercontinental breakup and a fertile metasedimentary crust was key in producing voluminous felsic volcanism via anatexis following the injection and emplacement of basaltic magmas into the lower crust.

**Supplementary Information:**

The online version contains supplementary material available at 10.1007/s00410-023-02065-1.

## Introduction

Silicic large igneous provinces are rare in Earth’s history. As such, they mark an exceptional convergence of conditions necessary to drive predominately explosive eruptions that produce large volumes of silicic magmas (e.g., Gianni and Navarrete 2022) and are intimately linked to supercontinent cycles throughout geologic history (Ernst 2014; Ernst et al. 2021). The Jurassic Chon Aike Silicic Large Igneous Province (CASP) consists of voluminous (ca. 235,000 km^3^; Pankhurst et al. 1998), crust-dominated felsic magmas generated during a period of widespread extension (Gust et al. 1985; Pankhurst and Rapela 1995; Pankhurst et al. 2000; Riley et al. 2001; Foley et al. 2023). The Jurassic magmatism and concurrent sedimentation occurred in response to the breakup of the Gondwanan supercontinent (Storey and Alabaster 1991; Riley and Leat 1999; Riley et al. 2017; Lovecchio et al. 2020); however, details on the geodynamic environment necessary to generate the observed volume of felsic magma remain under investigation (Navarrete et al. 2019; Bastias et al. 2021).

**Fig. 1:**
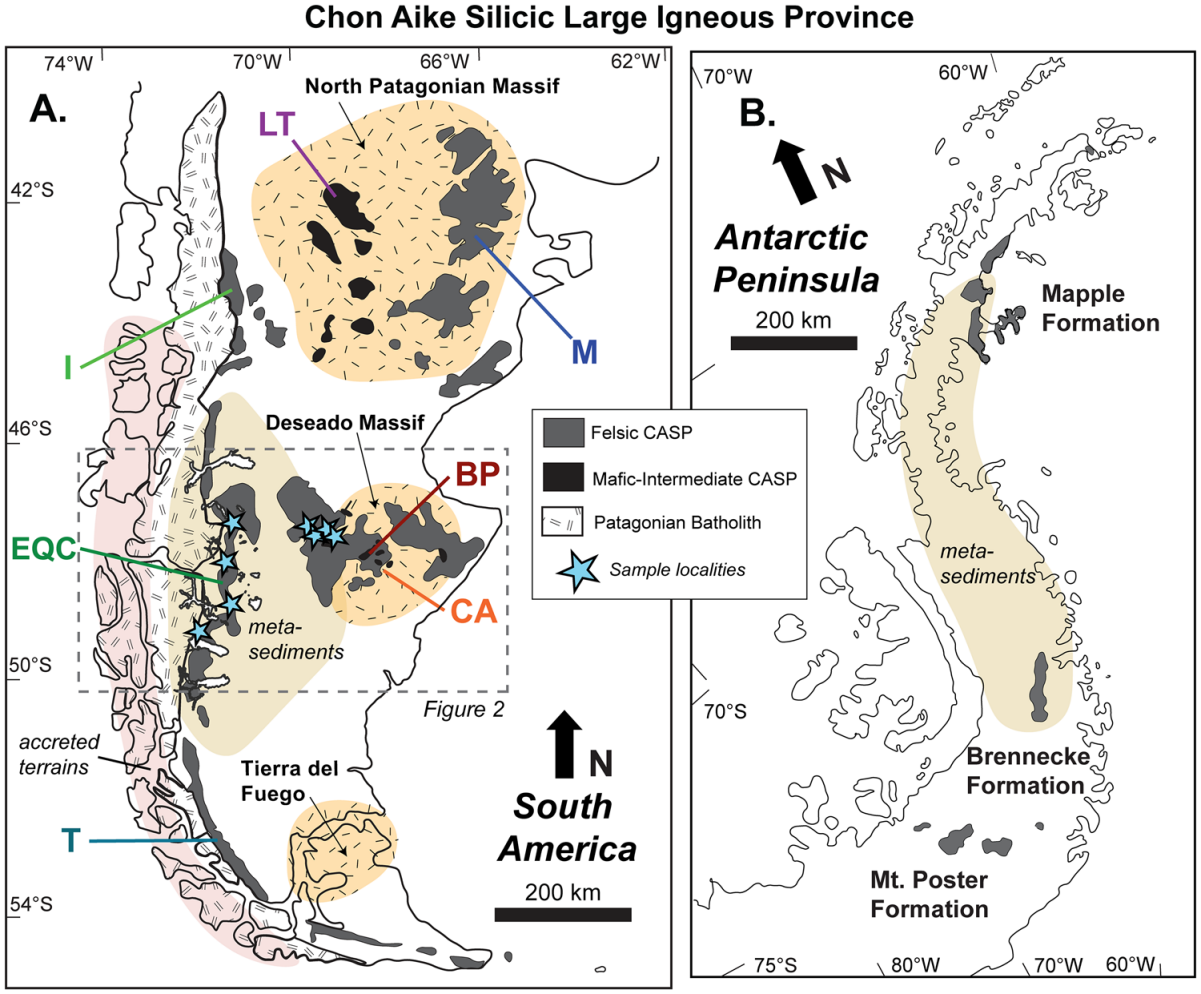
Simplified schematic of the Chon Aike Silicic Large Igneous Province (CASP) modified after Pankhurst et al. (2000), with outcrops in (**A**) Patagonia of South America and (**B**) the Antarctic Peninsula. Abbreviations for volcanic formations in Patagonia include *M* Marifil, *LT* Lonco Trapial, *I* Ibáñez, *EQC* El Quemado Complex, *CA* Chon Aike, *BP* Bajo Pobre, and *T* Tobífera

We focus on the Late Jurassic felsic volcanic units in the Patagonian CASP, including the El Quemado Complex (EQC) in the eastern Andean Cordillera and the western Chon Aike Formation (WCA) in the Deseado Massif (Fig. 1A). Previous isotope measurements of quartz δ^18^O compositions in these two units are among the highest values in the volcanic record (9–12‰; Foley et al. 2023). Considering that closed-system, mantle-derived rhyolites have δ^18^O melt values between 6.0 and 6.5‰ (Bindeman et al. 2004; Bindeman 2008), the generation of such high δ^18^O rhyolite compositions in the CASP requires a significant incorporation of crust-derived material. Such high δ^18^O values are typically found in rocks formed in low-temperature environments near or at the Earth’s surface. These include siliciclastic and argillic sediments and/or low-temperature, hydrothermally altered rocks (e.g., 10–30‰; Taylor 1980; Taylor and Sheppard 1986; Eiler 2001; Valley et al. 2005; Bindeman 2008). The incorporation of these materials can occur either by extensive assimilation of crustal rocks into mantle-derived melts or by crustal melting with a small contribution of melts from the mantle. Based on the ubiquity of widespread, voluminous high δ^18^O rhyolites, and their trace element compositions, the EQC and WCA were suggested to represent felsic magmas generated primarily by partial melting of metasedimentary basement lithologies (Foley et al. 2023).

**Fig. 2:**
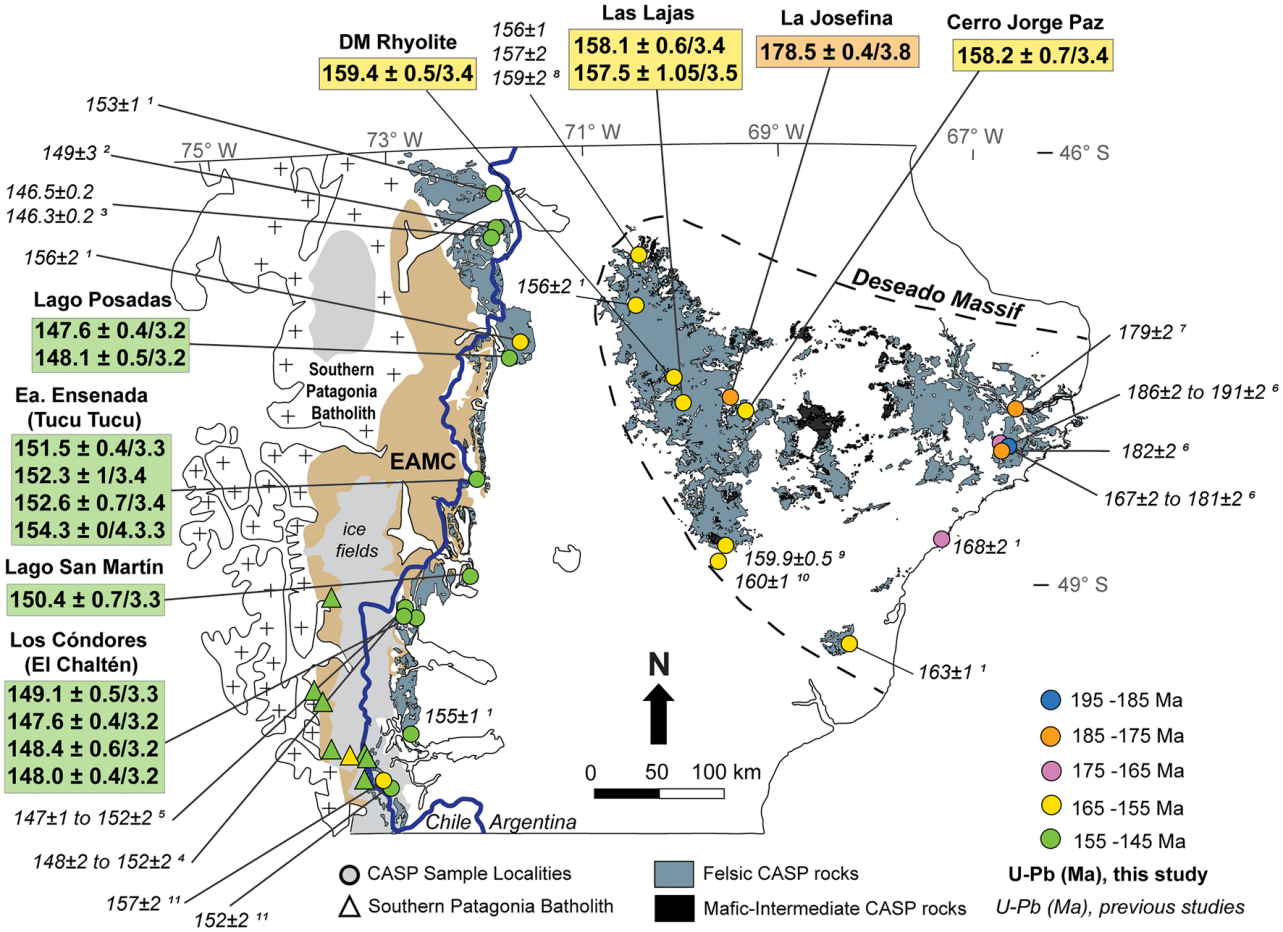
Geologic map displaying the volcanic rocks of the CASP, Southern Patagonian Batholith (SPB), and the Paleozoic Eastern Andean Metamorphic Complex (EAMC). Published zircon U–Pb ages for the CASP (circle symbols) are combined with the results of this study; ages are reported with 2 sigma errors. Ages of volcanism progress westerly across the region. Volcanism in the Chon Aike Formation is distinctly younger in the west than those in the east. The youngest volcanic units (EQC and Ibáñez Formations) are coeval with the oldest SPB granitoids in the Late Jurassic (triangle symbols). The border between Argentina and Chile is highlighted by a blue line. Numbered references include the following: (1) Pankhurst et al. (2000); (2) De la Cruz and Suarez (2006); (3) Poblete et al. 2013; (4) Malowski et al. 2016; (5) Foley et al. 2022; (6) Matthews et al. 2021; (7) Navarrete et al. 2020; (8) Permuy-Vidal et al. (2021); (9) Moreira et al. (2009); (10) Dube et al. (2003); (11) Hervé et al. (2007)

In this study, we present the first combined in situ analyses of U–Pb age data, together with trace element and O– and Lu–Hf isotope measurements from the EQC and WCA ignimbrites and some rhyolite flows to further constrain the age of the volcanic units as well as the geochemical characteristics of the source materials. We show that different source regions are required for the EQC and WCA, based on isotope compositions. The resistance of zircon grains to chemical alteration is important, since many ignimbrites and rhyolite flows in the CASP have been heavily affected by post-magmatic alteration, rendering the use of bulk-rock geochemical data difficult (Riley et al. 2001; Seitz et al. 2018; Foley et al. 2023). Moreover, the potential to combine U–Pb geochronology with Hf and O isotope tracers from a single zircon crystal is vital for constraining crustal evolution through time (e.g., Valley et al. 2005; Hawkesworth and Kemp 2006; Kemp et al. 2007; Vervoort and Kemp 2016). This is particularly important in southern Patagonia where its origin and evolution have remained elusive due to limited basement outcrops from the extended ice cover in the western Andes and burial from extensive volcanism associated with the CASP in the Deseado Massif (Fig. 2). In combination with isotope tracers, we use trace elements (e.g., Ti, U, and REEs) in zircon to constrain petrogenetic processes, including source rock types and conditions of crystallization for the CASP (e.g., Hanchar and Van Westrenen 2007; Grimes et al. 2015; Kirkland et al. 2015). Based on these results, we suggest a geodynamic framework for the generation of the voluminous, crust-dominated rhyolites at the southwest Gondwanan margin during the Late Jurassic.

## Geologic background

Due to the limited exposure of basement outcrops, the nature of the continental crust in Patagonia is not well known and has led to long-standing debates about its origin and relationship with the South American continent (Pankhurst et al. 2006; Ramos and Naipauer 2014; Calderón et al. 2016; González et al. 2018; Oriolo et al. 2023). For units that are exposed, the investigated outcrops of basement rocks in Patagonia record a long-lived history of a convergent margin, with episodes of extension and rifting, as well as terrane accretion that is detailed in a series of orogenic phases since the Cambrian (Forsythe 1982; Ramos 1988; Suárez et al. 2019a; Calderón et al. 2020). To trace potential crustal sources for the felsic magmas generated during the Jurassic, we present a summary of basement lithologies associated with the volcanic units studied here.

The igneous basement exposures in southern Patagonia are confined to the eastern half of the Deseado Massif and range in age from Late Neoproterozoic to Triassic (Rapela and Pankhurst 1996; Pankhurst et al. 2003; Navarrete et al. 2019), while, toward the west, the basement rocks are predominately metasedimentary in origin (Fig. 1A). In the western Deseado Massif, the Paleozoic metasedimentary units of the La Modesta and Cerro Negro Formations comprise shallow marine sediments deposited within a forearc/foreland basin along an active mid-Silurian volcanic arc which is represented by exposed granitoids in the eastern Deseado Massif (Guido et al. 2004; Moreira et al. 2013; Permuy-Vidal et al. 2014; Suárez et al. 2019a). The low-grade metamorphosed protoliths of La Modesta Formation are composed of alternating pelitic, metavolcanics, calc-silicates, and graywacke lithologies (Moreira et al. 2005, 2013). Arc migration and the progression of sediment deposition trends westwards, indicated by the decrease in maximum sedimentation ages in the Eastern Andean Metamorphic Complex (EAMC) (ca. 383–240 Ma; Hervé et al. 2003; Augustsson et al. 2006). Locally in the region of Argentina associated with the EQC, the EAMC is referred to as the Bahía de la Lancha Formation and is dominated by low-grade metasediments of turbidite sequences (Riccardi 1971).

During the Jurassic, the outpouring of volcanic rocks associated with the Chon Aike Silicic Large Igneous Province covered ~ 1,000,000 km^2^ across Patagonia and the Antarctic Peninsula (Fig. 1), with an estimated volume of 235,000 km^3^ (Pankhurst et al. 1998). The extrusive units are primarily felsic in composition (> 70 wt. % bulk SiO_2_) and consist of ignimbrite flows with minor rhyolitic domes, ash flows and falls, and subvolcanic deposits (e.g., Pankhurst et al. 1998; Guido 2004; Moreira et al. 2014; Navarrete et al. 2020; Foley et al. 2022, 2023). Mafic to intermediate rocks in the CASP are minor, occupying an exposed area of ca. 50,000 km^2^. Many CASP formations consist of volcanic units which have a bimodal distribution between rhyolite and andesite compositions (e.g., Gust et al. 1985; Pankhurst and Rapela 1995; Riley et al. 2001; Matthews et al. 2021).

Previously determined U–Pb zircon crystallization ages in the CASP range from ca. 193 Ma in the Northern Patagonian Massif (e.g., Pavón-Pivetta et al. 2020) to the youngest along the eastern Andean margin at ca. 146 Ma (e.g., Poblete et al. 2013). In Patagonia, the oldest phase of felsic magmatism began in the northeast of the North Patagonian Massif with the Marifil Formation at ca. 180–190 Ma (Pankhurst and Rapela 1995; Pankhurst et al. 2000; Strazzere et al. 2018, 2021; Pavón-Pivetta et al. 2020; Falco et al. 2022) (Fig. 1A). Magmatism continued to the west with primarily mafic volcanism and U–Pb ages ranging from ca. 170–188 Ma in the Lonco Trapial Formation (Franzese et al. 2006; Cúneo et al. 2013; Bouhier et al. 2017; Hauser et al. 2017; Zaffarana et al. 2020).

Recent U–Pb ages measured in the ignimbrites of eastern Deseado Massif extend the duration of the Chon Aike Formation to 30 Myr (Matthews et al. 2021) (Fig. 2). The older ages of ca. 191 Ma correlate in time and composition with the bimodal volcanism in the North Patagonian Massif. From 180 until 167 Ma, volcanism is dominated by felsic compositions in the eastern region of the Deseado Massif (Pankhurst et al. 2000; Navarrete et al. 2020; Matthews et al. 2021). While in the western region, the Chon Aike Formation volcanic units are distinctly younger at ca. 160–156 Ma (Dube et al. 2003; Moreira et al. 2009; Permuy-Vidal et al. 2021) (Fig. 2).

The youngest felsic formations follow along the eastern Andean margin and include the El Quemado Complex (EQC) and its equivalent formations in Chile, the Ibañez and Tobífera Formations (Fig. 1A). Ages for the EQC and Ibañez Formations are reported between 157 and 146 Ma (Pankhurst et al. 2000; Rolando et al. 2004; Poblete et al. 2013; Malkowski et al. 2016; Foley et al. 2022) (Fig. 2). The earliest published age for the Tobífera Formation is 172 ± 1 Ma as determined from a borehole sampled in Tierra del Fuego (Pankhurst et al. 2000); however, more recent U–Pb ages of ca. 149 and 160 Ma were reported for the Tobífera Formation outcropping within the Andes (Calderón et al. 2007; Muller et al. 2021).

In the Antarctic Peninsula, the felsic Mount Poster and Brennecke Formations are coeval with the oldest episodes of Patagonian magmatism at ca.184 to 189 Ma (Pankhurst et al. 2000) (Fig. 1B), while the Maple Formation is distinctly younger, ranging from 173 to 163 Ma (Pankhurst et al. 2000; Scasso et al. 2022).

## Methods

### Studied volcanic samples and zircon preparation

Zircon grains from 16 samples were separated from felsic volcanic rocks of the El Quemado Complex (EQC) and the western Chon Aike Formation (WCA) that have been previously characterized by Foley et al. (2023). All zircon separated from the EQC (*n* = 11) are from bulk ignimbrite samples, while the WCA (*n* = 5) includes three ignimbrite samples and two rhyolitic lava samples (Table 1). We sampled the EQC at four locations along a north–south ~ 230 km transect (Fig. 2) that include ignimbrite profiles at Lago Posadas, Estancia (Ea.), Ensenada (Tucu Tucu), and at Los Cóndores (El Chaltén), and a single ignimbrite from the fourth locality at Lago San Martín (Table 1). For each of the three ignimbrite profiles, zircon grains were separated from both the uppermost and lowermost ignimbrite units. At both Ea. Ensenada and Los Cóndores localities, additional ignimbrites were sampled within the sequence. In the western Deseado Massif, we sampled four different eruptive units from the WCA across a west to east profile that include a rhyolitic flow (Deseado Massif rhyolite), the Las Lajas ignimbrite, the La Josefina Dome, and the Cerro Jorge Paz ignimbrite (Fig. 2).

**Table 1:** Summary of U–Pb average zircon crystallization age with associated MSWD and number of analyses used

Sample	Formation-Unit	^206^Pb/^238^U age (Ma)	± 2 S.D. Minimum (95% c.l.)/maximum (extended error)	MSWD	Analyses used in age determination	Inherited zircon ^206^Pb/^238^U date (Ma); *^207^Pb/^206^Pb (Ma)
LPMF1	EQC-Lago Posadas (Ign A)	**147.6**	**0.4/3.2**	1.5	17	464 ± 6; 513 ± 6
LPMF10	EQC-Lago Posadas (Ign G)	**148.1**	**0.45/3.2**	0.66	12	576 ± 7
EEMF10	EQC-Ea. Ensenada (Ign A)	**151.5**	**0.41/3.3**	2.3	18	
EEMF11	EQC-Ea. Ensenada (Ign B)	**152.3**	**1/3.4**	2.2	7	
EEMF9	EQC-Ea. Ensenada (Ign E)	**152.6**	**0.7/3.4**	0.81	8	213 ± 10; 523 ± 12
EEMF4	EQC-Ea. Ensenada (volcanoclastic)	**154.3**	**0.4/3.3**	1.1	11	634 ± 6; 2756 ± 18*
LSMMF1	EQC-Lago San Martín	**150.4**	**0.7/3.3**	1.8	17	544 ± 8
LCMF12	EQC-Los Cóndores (Ign A)	**149.1**	**0.5/3.3**	1.4	16	336 ± 3; 497 ± 5; 634 ± 6
LCMF4	EQC-Los Cóndores (Ign F)	**147.6**	**0.4/3.2**	0.81	22	344 ± 3; 357 ± 6; 1331 ± 24 (1368 ± 43*)
LCMF1	EQC-Los Cóndores (Ign G)	**148.4**	**0.6/3.2**	1.6	32	185 ± 2; 233 ± 5; 275 ± 3; 283 ± 4; 318 ± 11; 374 ± 6; 503 ± 12
LCMF14	EQC-Los Cóndores (Ign H)	**148**	**0.4/3.2**	1.2	40	171 ± 4; 202 ± 2; 240 ± 6; 246 ± 4; 285 ± 3; 294 ± 7; 346 ± 8; 366 ± 8; 455 ± 12; 500 ± 5; 506 ± 11; 552 ± 6; 615 ± 8; 972 ± 42*; 1014 ± 20 (1068 ± 114*); 1878 ± 30*
DMMF1	WCA-Deseado Massif rhyolite dome	**159.4**	**0.5/3.4**	0.6	11	
LLMF3	WCA-Las Lajas ignimbrite (densely welded)	**158.1**	**0.6/3.4**	1.3	12	
LLMF6	WCA-Las Lajas ignimbrite (poorly welded)	**157.5**	**1.05/3.5**	1.8	13	
LMJMF3	WCA-La Josefina rhyolite Dome	**178.5**	**0.4/3.8**	0.5	5	
DMMF3	WCA-Cerro Jorge Paz ignimbrite	**158.2**	**0.7/3.4**	1.1	13	1254 ± 40*

The list of concordant xenocrystic zircon dates are organized by sample name and locality

Hand samples were coarsely crushed to a size fraction < 500 µm using a hydraulic press. Samples were further crushed using a disk-mill until equal proportions of size fractions ranging between 250 < *x* < 125 µm, 125 < *x* < 90 µm, and < 90 µm were obtained. Zircon separates were selected for analysis from both size fractions of 125–250 and 90–125 µm. Phenocrysts of zircon were mounted in epoxy and polished down to obtain a section closest to the center of the crystals. The surfaces of epoxy mounts were checked using a Zeiss White Light Interferometer to ensure a polishing quality which resulted in less than 5 microns of topography to minimize the effects of topography on oxygen isotope measurements during the SIMS sessions (e.g., Kita et al. 2009). Zircon grains were then imaged on a scanning electron microscope at a low resolution to target potential areas of interest for SIMS analysis using a cathodoluminescence (CL) detector attached to a CamScan MV2300 Scanning Electron Microscope. The zircon grains were imaged using an accelerating voltage of ~ 10 kV and current of < 1.0 nA. Low-resolution and low-current images were acquired prior to the SIMS session to minimize the damage induced by the electron beam to the epoxy around zircon grains. Following the SIMS analyses, high-resolution images of zircon grains using longer dwell times were acquired to document the placement of in situ measurements (SIMS and LA-ICP-MS; see below).

### Secondary ion mass spectrometry

In situ measurements of ^18^O/^16^O ratios were conducted using the Secondary Ion Mass Spectrometer (SIMS) at the WiscSIMS facility of the University of Wisconsin in Madison, Wisconsin (USA) using a Cameca IMS 1280 (10 out of 14 samples; Supplementary T1–T2). Four samples were also analyzed at the SwissSIMS facility using the Cameca IMS 1280-HR at the University of Lausanne (Switzerland). The measurements followed the description of Kita et al. (2009) with a 10 kV Cs + primary beam and a ~ 2 nA current, resulting in a beam size of ~ 10 µm. A normal incidence electron gun was used to compensate for charging on the sample surface and tuned at the beginning of each session. The intensities of ^18^O and ^16^O ions were analyzed simultaneously on Faraday cups in a multicollection mode with a mass resolving power (MRP) of ~ 2200. In addition, for the session at the WiscSIMS laboratory, to check for radiation damage, ^16^O^1^H intensities were measured on the mono-collection faraday cups with a MRP of 5000 to avoid ^17^O interference. Faraday cup backgrounds were calibrated approximately every 12 h using Cameca’s software. Each analysis consisted of pre-sputtering (~ 30 s), automated centering of secondary deflectors (~ 60 s), and 16 cycles of 4 s counting times. Because the primary beam density was very high, samples analyzed at the WiscSIMS laboratory exhibited a mass-dependent fractionation of the ^18^O/^16^O ratios during each individual analysis, i.e., ^18^O/^16^O ratios were decreasing over the time of an individual analysis. Similar to what was described in Foley et al. (2023) to correct for the mass-dependent fractionation, a linear regression was fitted for ^18^O/^16^O ratios versus cycle numbers for every standard analysis, and then an average slope was used to correct for all analyses. To correct for instrumental bias, the Penglai zircon standard was used as the internal reference standard for all sessions and has an average δ^18^O of 5.31 ± 0.10 ‰ (Li et al. 2010). To monitor for potential drift over time during the sessions, two to four unknown analyses were bracketed by at least one analysis of Penglai. No drift was observed for the different sessions. The ^18^O/^16^O ratios were normalized relative to the Vienna Standard Mean Ocean Water (VSMOW).

Final uncertainty on individual SIMS δ^18^O measurements was computed through the propagation of internal error (2SE), reproducibility of standard analyses used to calibrate the unknown analysis (2SE), and the uncertainty on the reference value of Penglai (1SD). Typical reproducibility (2SD) of repeated Penglai analyses was 0.15–0.3‰.

### Laser ablation Inductively coupled plasma Mass spectrometry

**Fig. 3:**
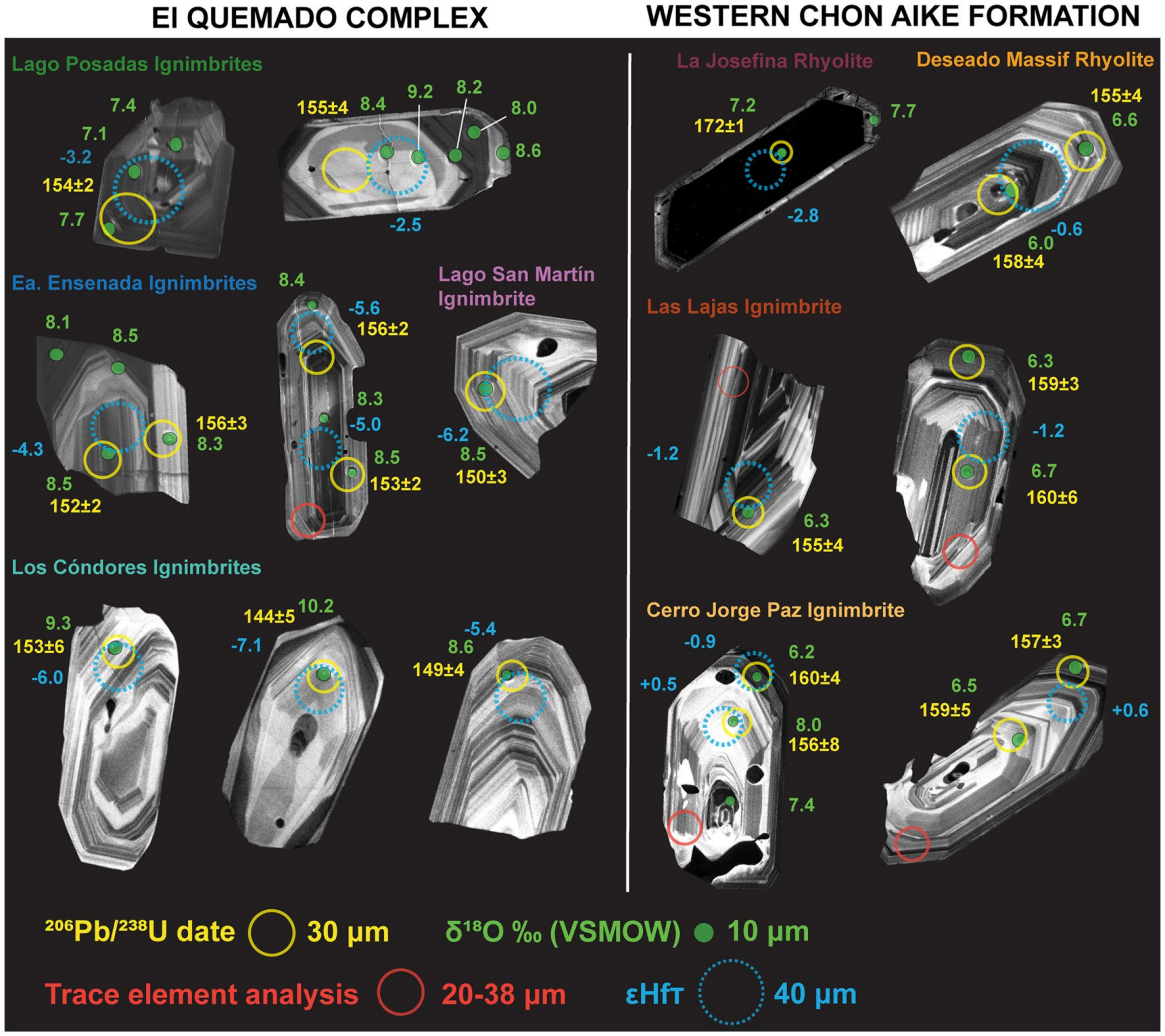
Characteristic zircon cathodoluminescence patterns for the EQC and WCA volcanic rocks displayed with the corresponding values of δ^18^O, εHf_T_, and U–Pb date. Many of the EQC zircon display fine-scale oscillatory zonation with subtle changes in grayscale variation, while the WCA zircon have much greater variability in CL textures and grayscale intensities. The La Josefina rhyolite zircon are consistently black in CL which corroborates with the extremely high Uranium concentrations measured

**Fig. 4:**
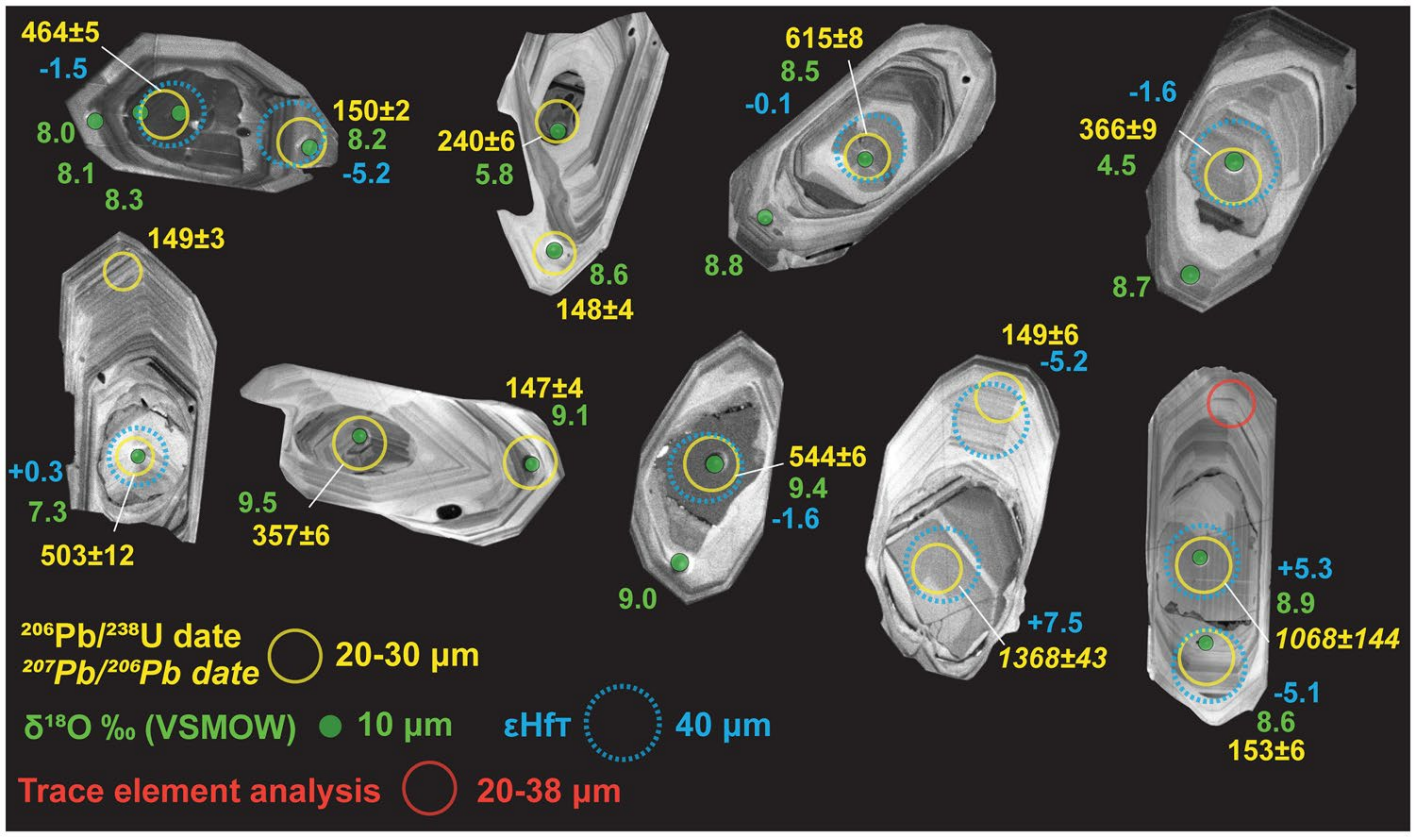
Inherited zircon preserved in the EQC with commonly thick overgrowths of Jurassic rims. Cathodoluminescence (CL) imaging reveals a variety of inherited zircon textures and sizes, but most cores show small-scale (< 1 μm) rhythmic oscillations, consistent with a magmatic origin. Few cores are homogeneous in CL intensity and are more indicative of metamorphic growth textures. The thicknesses of the Jurassic overgrowths vary but are often > 40 μm (parallel to the c-axis), suggesting that incorporation of the inherited zircon grains occurred at the source, as opposed to late-stage contamination via upper-crustal assimilation. Isotopic compositions of the inherited cores are variable, but oxygen values are commonly high (> 7‰) indicating isotope compositions which reflect a paleo-record of crustal reworking

Following the SIMS measurements, laser ablation inductively coupled plasma mass spectrometry (LA-ICP-MS) U–Pb analyses were performed using a Thermo Fisher Scientific ELEMENT XR sector-field ICP-MS interfaced to a RESOlution SE 193 nm ArF excimer laser ablation system at the Institute of Earth Sciences, UNIL (Supplementary T3–T5). The methods are described in detail in Ulianov et al. (2012). The ablation system was operated using spot sizes of 20 or 30 µm, with a repetition rate of 5 Hz and fluence of 3 J/cm^2^. For larger grains, a spot size of 38 µm was used. When possible, U–Pb spots were placed such that they overlapped with SIMS analyses (Figs. 3 and 4). Helium was used as the carrier gas. Natural reference zircon GEMOC GJ-1, with a reported CA-ID-TIMS ^206^Pb/^238^U age of 600.28 ± 0.16 (Schaltegger et al. 2021), was used as a primary standard for the determination of the relative sensitivity factors. Natural reference zircon Plešovice was used as a secondary standard for accuracy control (337.207 ± 0.029 Ma; Sláma et al. 2008; Widmann et al. 2019). The isotope homogeneity of the ablated material was monitored on time-resolved ^206^Pb/^238^U and ^207^Pb/^235^U spectra, and analyses with irregularly changing ratios through time were interpreted to reflect mixing between different age domains or isotopic disturbance. Such measurements were discarded. Data reduction is carried out using LAMTRACE (Jackson 2008). The U–Pb data are not corrected for common Pb and any analysis with concentrations of enriched ^204^Pb is excluded from calculation of the weighted mean sample age. Only concordant analyses are included in the calculation of weighted mean sample age (Horn et al. 2000; Gerdes and Zeh 2006). Concordia diagrams and weighted mean crystallization ages are calculated using IsoplotR version 4.3 (Vermeesch 2018) (Supplementary F1–3). Given the excess scatter in all dated rocks, even after removal of anomalously old or young analyses, the reported weighted mean ages of the selected population of analyses may not be accurate within uncertainty determined purely from data scatter. In addition to the error calculated in IsoplotR that we take as a minimum error, we follow the method of Ruiz et al. (2022) in calculating an extended error. This extended error, even though probably overestimated, allows for a more accurate age interpretation from scattering U–Pb dates, which is more important than apparent precision. We report this as an extended error in the format [X] (95% c.l.) / [Y] (extended error) in Table 1.

The reproducibility of GJ-1 in all sessions was 0.60 to 0.90% (2 S.D.). In each session, the secondary standard Plešovice gave a weighted mean ^206^Pb/^238^U age that was statistically indistinguishable from its nominal value once the error propagation was performed (Supplementary T4). Over five analytical sessions, a total of 19 analyses (out of 164) of the secondary standards were excluded, 16 for being discordant and three whose ages were clear outliers.

The Lu–Hf isotope measurements were carried out after SIMS δ^18^O and LA-ICP-MS U–Pb geochronology measurements (Supplementary T6–T8). The Lu–Hf measurements were carried out at the University of Geneva (Switzerland) on a Thermo Neptune Plus MC-ICP-MS connected to a Teledyne-Photon Machines Analyte G2 193 nm ArF excimer laser system, equipped with a two-volume HelEx-2 ablation cell (d’Abzac et al. 2014). The ablation was performed at a fluence of ~ 4 J/cm^2^, repetition rate of 5 Hz, and spot size of 40 μm; spots were placed such that they partially overlap with U–Pb analyses, when possible. Helium was used as a carrier gas for the ablated particles and mixed with a small amount of N_2_ before entering the Ar plasma torch to increase sensitivity. Measurements were performed with a low mass resolution over 120 cycles of ~ 1 s; standard grains were ablated for a total of 120 cycles of a duration of 1 s each and the measurements ranged between 20 and 120 cycles, depending on the thickness of the zircon grains (> 80% of analysis range between 60 and 120 cycles). Each series of ~ 15–20 unknown sample measurements was bracketed by measurements of a blank, Mud Tank (^176^Hf/^177^Hf 0.282507 ± 0.000006 2SD; Woodhead and Hergt 2005; Gain et al. 2019), Plešovice (^176^Hf/^177^Hf 0.282482 ± 0.000013 2SD; Sláma et al. 2008), MUN4 (^176^Hf/^177^Hf 0.282135 ± 0.000007; Fisher et al. 2011), and GJ-1 (^176^Hf/^177^Hf 0.282000 ± 0.000005; Morel et al. 2008) zircon standards. The four standards are measured and evaluated in real time during the analysis to evaluate the offset of the measured values to reference values. Blanks were acquired for the full 120 cycles (without ablation) and for the same time interval as the zircon standard measurements.

Data were reduced off-line using an excel spreadsheet and consisted of blank subtractions, removing the isobaric interference of ^176^Lu and ^176^Yb on mass 176 (e.g., Fisher et al. 2011) and correcting the resulting ^176^Hf/^177^Hf ratio for mass bias using an exponential law (Albarède et al. 2004). βHf and βYb mass bias coefficients were calculated from the measured ^179^Hf/^177^Hf and ^173^Yb/^171^Yb with the reference values of Patchett and Tatsumoto (1981) (^179^Hf/^177^Hf = 0.7325) and Thirlwall and Anczkiewicz (2004) (^173^Yb/^171^Yb = 1.1234), respectively. Isobaric interferences of ^176^Yb and ^176^Lu with ^176^Hf were corrected using ^176^Yb/^173^Yb = 0.786954 and ^176^Lu/^175^Lu = 0.02645 respectively (Thirlwall and Anczkiewicz 2004). Only non-perturbed spectra were retained. Initial ^176^Hf/^177^Hf ratios and epsilon notation (εHf_T_) values are calculated using the LA-ICP-MS ^206^Pb/^238^U age of the corresponding crystal and the CHUR parameters of Bouvier et al. (2008) (^176^Hf/^177^Hf = 0.282785 and ^176^Lu/^177^Hf = 0.0336) and λ^176^Lu = 1.87 × 10^–11^/year (Söderlund et al. 2004).

Trace elements in zircon were determined using the Thermo ELEMENT XR sector-field ICP-MS interfaced to the RESOlution SE 193 nm excimer laser ablation system that was also used for the U–Pb dating of zircon (Supplementary T9-T10). Ablation settings included a fluence of 5 J cm^−2^ with a 10 Hz repetition rate and beam diameters ranging between 20 and 38 μm, using the maximum size when possible to maximize the net signal. ^27^Al, ^42^Ca, ^88^Sr, and ^137^Ba were analyzed to screen for analyses that correspond to alteration or mineral inclusions, in addition to ^29^Si, ^45^Sc, ^49^Ti, ^89^Y, ^91^Zr, ^93^Nb, ^139^La, ^140^Ce, ^141^Pr, ^143^Nd, ^147^Sm, ^151^Eu, ^157^Gd, ^159^ Tb, ^163^Dy, ^165^Ho, ^166^Er, ^169^Tm, ^172^Yb, ^175^Lu, ^178^Hf, ^181^Ta, ^232^Th, and ^238^U. Measurements were placed next to the SIMS spots for maximum correlation between the O isotope and trace element concentration data. SRM612 was employed as the primary standard; BCR-2G glass was measured along with unknown zircon grains and used as the secondary standard for quality control. Data reduction was conducted using LAMTRACE (Jackson 2008).

## Results

We first report the results of SEM-CL imaging, followed by the radiogenic (U–Pb, Lu–Hf) and O isotope measurements carried out within single zircon crystals. It is noted that zircon analyses have the corresponding quartz δ^18^O data in Foley et al. (2023) that allow for the calculation of mineral pair isotope equilibrium temperatures. Zircon and quartz pairs were analyzed from the same hand sample, except for the Deseado Massif rhyolite flow where quartz and zircon grains were analyzed from different samples in the same flow (quartz from sample DMMF2 and zircon from DMMF1).

We distinguish zircon populations as either *Jurassic autocrystic* grains*,* which are thought to have directly precipitated from the host melt, or as older *inherited* grains derived from a contributing source material and/or entrained via assimilation-related contamination.

### Zircon morphology

#### Jurassic autocrystic zircon

Zircon phenocrysts range in morphology from long and prismatic (parallel to the c-axis) to rounded and short (Fig. 3). Many zircon grains are euhedral and have width-to-length ratios between ~ 1:2 and 1:4. There is typically a subset of the zircon population with an aspect ratio of up to 1:10.

Cathodoluminescence (CL) imaging of hundreds of zircon grains per sample reveals large variability in morphology and texture within each sample and across the volcanic units studied (Fig. 3). Many of the EQC autocrystic zircon have fine-scale oscillatory zonation, with subtle variations in grayscale intensity (Fig. 3). The WCA zircon has a larger variability in CL textures between the different units studied. The zircon grains of the La Josefina Dome are consistently homogeneously black with thin (< 10 μm) gray rims (Fig. 3). Many WCA zircon grains are characterized by oscillatory zonation, though individual growth bands are significantly darker (to black) on average when compared to the EQC zircon. Inclusions are present in zircon of both the EQC and WCA, with apatite inclusions the most abundant in the WCA.

#### Inherited zircon

Multiple inherited cores were identified based on distinct changes in CL textures (Fig. 4); growth surfaces are truncated by dissolution surfaces that are then overgrown by the euhedral, oscillatory growth zones of magmatic zircon. Inherited cores have CL patterns that range from homogeneous to oscillatory. The sizes of inherited cores range from < 20 μm to ~ 75 μm. Similarly, the range in length scale of overgrowth varies between individual grains, from the smallest at < 20 μm (c-axis) to thicker overgrowths of ~ 80 μm (Fig. 4).

### U–Pb geochronology

A summary of the 750 U–Pb spot analyses, including the ^206^Pb/^238^U average zircon crystallization ages, the number of analyses used for each age determination, and dates of inherited zircon cores for each sample, is presented in Table 1. Inherited zircon cores are listed with their corresponding ^206^Pb/^238^U date. For zircon older than 900 Ma, the ^207^Pb/^206^Pb dates are also indicated (Supplementary T5), as it is the more accurate chronometer for older grains.

The mean weighted LA-ICP-MS U–Pb zircon crystallization age for each volcanic unit is calculated using the largest plateau of concordant analyses that overlap within uncertainty (Supplementary F3). For the samples dated here, our preferred interpretation does not significantly alter the calculated age of the volcanic unit, but it does narrow the final uncertainty associated with the age.

#### Zircon crystallization ages: El Quemado Complex

The order of descriptions for the four localities sampled for the EQC follows the 230 km north–south transect along the eastern Andean margin (Fig. 2). The two ignimbrites at Lago Posadas, sampled from the upper- and lowermost portion of the sequence have similar average ages at 147.6 ± 0.4/3.2 and 148.1 ± 0.45/3.2 Ma, respectively (Table 1). Further south, the weighted mean zircon ^206^Pb/^238^U ages for the three ignimbrites at Estancia (Ea.) Ensenada (Tucu Tucu) range from 151.5 ± 0.41/3.3 Ma in the uppermost capping ignimbrite (EEMF10) to 152.6 ± 0.7/3.4 Ma in the lowermost ignimbrite (EEMF9). The zircon crystallization age determined for a third ignimbrite sampled below the uppermost ignimbrite unit is 152.3 ± 1/3.4 Ma (EEMF11). The fourth sample taken from within an interlayered volcanoclastic sequence underlying the main ignimbrite sequence was dated at 154.3 ± 0.4/3.3 Ma (EEMF4). At Lago San Martin, the average zircon crystallization age for an ignimbrite is 150.4 ± 0.7/3.3 Ma (LSMMF1). At the southernmost locality sampled for the EQC, four ignimbrites were dated from the 220 m thick sequence at the Los Cóndores wall in El Chaltén. The average zircon crystallization ages for both the uppermost (LCMF12) and lowermost ignimbrite (LCMF1) units are almost identical at 149.1 ± 0.5/3.3 and 148.4 ± 0.6/3.2 Ma. An additional ignimbrite that overlies the lowermost ignimbrite (LCMF1) was dated at 147.6 ± 0.4/3.2 Ma (LCMF4). The fourth ignimbrite (LCMF14) was sampled along the Los Cóndores wall, but to the north of the main sample profile. The average zircon crystallization age is also 148 ± 0.4/3.2 Ma.

Combined, the ^206^Pb/^238^U zircon crystallization ages for the EQC ignimbrites range from 148 to 153 Ma (Supplementary F1), with most of the dates clustering around 150 Ma. The northern and southernmost localities are similar in age at 148 to 149 Ma, while the Ea. Ensenada section is slightly older. At the three locations of Lago Posadas, Ea. Ensenada, and El Chaltén, upper and lower ignimbrites have average crystallization ages that are within error (Table 1).

#### Zircon crystallization ages: western Chon Aike Formation (Deseado Massif)

Concordant ^206^Pb/^238^U dates for four WCA samples range from 144 to 167 Ma (*n* = 88), with the weighted mean ^206^Pb/^238^U ages ranging from 157.5 to 159 Ma (Table 1, Supplementary F1). There is no spatial trend in the average crystallization age, with the westernmost rhyolitic dome (DM rhyolite) and the easternmost sample (Cerro Jorge Paz ignimbrite) overlaps within uncertainty with weighted mean ages of 159.4 ± 0.5/3.4 (DMMF1) and 158.2 ± 0.7/3.4 (DMMF3). The two samples of the Las Lajas ignimbrite slightly differ in age, with the youngest age, 157.5 ± 1.05/3.5 Ma (LLMF6), determined for the poorly welded portion of the ignimbrite and 158.1 ± 0.6/3.4 Ma (LLMF3) for the densely welded portion of the ignimbrite.

**Fig. 5:**
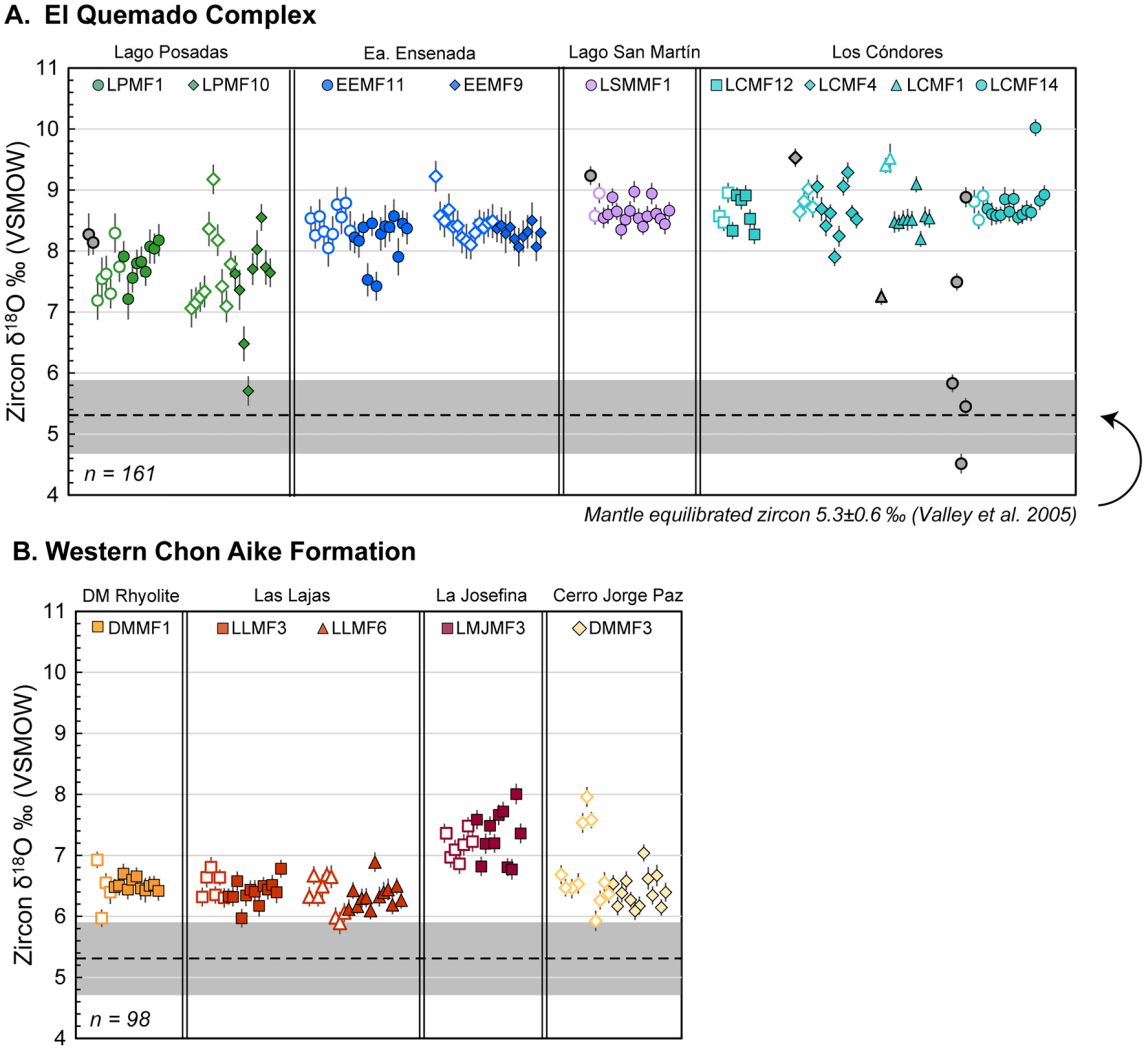
SIMS oxygen isotope analyses of EQC and WCA zircon. Oxygen isotope values are ordered by sample and analysis sequence. Open symbols indicate core analyses, whereas closed symbols indicate rim analyses. Gray symbols represent an analysis within an inherited zircon core from the respective sample. Sample populations are relatively homogeneous in their δ^18^O value, apart from LPMF10 with two outlier rim compositions of < 7‰. EQC zircon values are 1–2‰ greater than those of the WCA, though all zircon values are elevated relative to the mantle equilibrated zircon oxygen isotope value of 5.3 ± 0.6‰ (Valley et al. 2005). The EQC zircon consistently records some of the highest measured δ^18^O value for igneous zircon, when compared to worldwide compilations of zircon compositions (e.g., Valley et al. 2005; Cavosie et al. 2011)

The rhyolite dome at La Josefina (LMJMF3) is the oldest unit dated in this study, with an average zircon crystallization age of 178.5 ± 0.4/3.8 Ma. This average crystallization age was determined with the largest plateau of overlapping analyses (Fig. 5B), but concordant analyses range from 168 to 181 Ma.

#### Inherited zircon dates

Multiple concordant inherited zircon cores were analyzed within the EQC zircon with overall dates ranging from 171 to 2756 Ma (*n* = 37; Table 1). At Lago Posadas, concordant inherited dates include 464 ± 5 and 513 ± 4 Ma from the uppermost ignimbrite [LPMF1] (Fig. 4) and a single concordant inherited core in the lowermost [LPMF10] ignimbrite yielded a date of 567 ± 5 Ma. At Ea. Ensenada, two inherited cores at 213 ± 10 and 523 ± 11 Ma were analyzed in an upper unit [EEMF9], while the lowermost unit [EEMF4] yielded two older dates of 634 ± 4 Ma (^206^Pb/^238^U) and 2697 ± 8 Ma (^207^Pb/^206^Pb), which is the oldest zircon core analyzed in this study (Fig. 4). A single date of 544 ± 6 Ma was analyzed within LSMMF1 (Fig. 4). The four Los Cóndores ignimbrite samples have more inherited zircon cores than all other EQC localities, with the LCMF14 ignimbrite encompassing most of the inherited dates with 16 out of 29 analyses with ages ranging from Late Triassic to oldest Paleoproterozoic (Table 1).

Five inherited cores were analyzed from each of the WCA volcanic rock sampled, but surprisingly, only one analysis was concordant in the Cerro Jorge Paz ignimbrite at 1254 ± 40 Ma (^207^Pb/^206^Pb) (Supplementary T5).

### Oxygen and hafnium isotope geochemistry

The Lu–Hf systematics of the studied samples is presented as the initial εHf_T_ values, with respect to the CHUR, based on the corresponding ^206^Pb/^238^U crystallization age measured from the same zircon domain; if a zircon yielded a discordant U–Pb analysis, the average zircon crystallization age of the sample was used (Supplementary T8). Because Hf isotope values vary systematically with δ^18^O values in the EQC and WCA, the isotope compositions are presented together below.

#### Jurassic autocrystic zircon from the EQC

The EQC zircon δ^18^O values (*n* = 161) cover a range from 5.7 to 10.0‰, with all but three values clustering in the narrow range from 7.1 to 9.5‰ (Fig. 5A). In fact, 70% of the oxygen isotope compositions from the EQC zircon are between 8 and 9‰ (*n* = 113). Measurements from core to rim in the same zircon typically do not exhibit significant intra-grain compositional variations, even in instances where complex CL zonation is observed (e.g., Lago Posadas and Ea. Ensenada; Fig. 3). Many of the zircon analyses have very consistent δ^18^O values for a single grain, within the analytical uncertainty of ± 0.2‰ (Supplementary T2). A few outliers in the EQC data are attributed to inherited cores (see below), which have variable oxygen isotope compositions and have significantly older U–Pb dates (gray symbols, Fig. 5A). Notably, the two ignimbrites from Lago Posadas have the lowest δ^18^O values (lowest value of 5.7‰) and more isotopic inter-grain variability than the other three EQC localities. The zircon of LPMF10 have the largest oxygen isotope variability for all units studied; autocrystic cores range from 7.1 to 9.2‰, while rim analyses range from 5.7 to 8.6‰ (Fig. 5A).

**Fig. 6:**
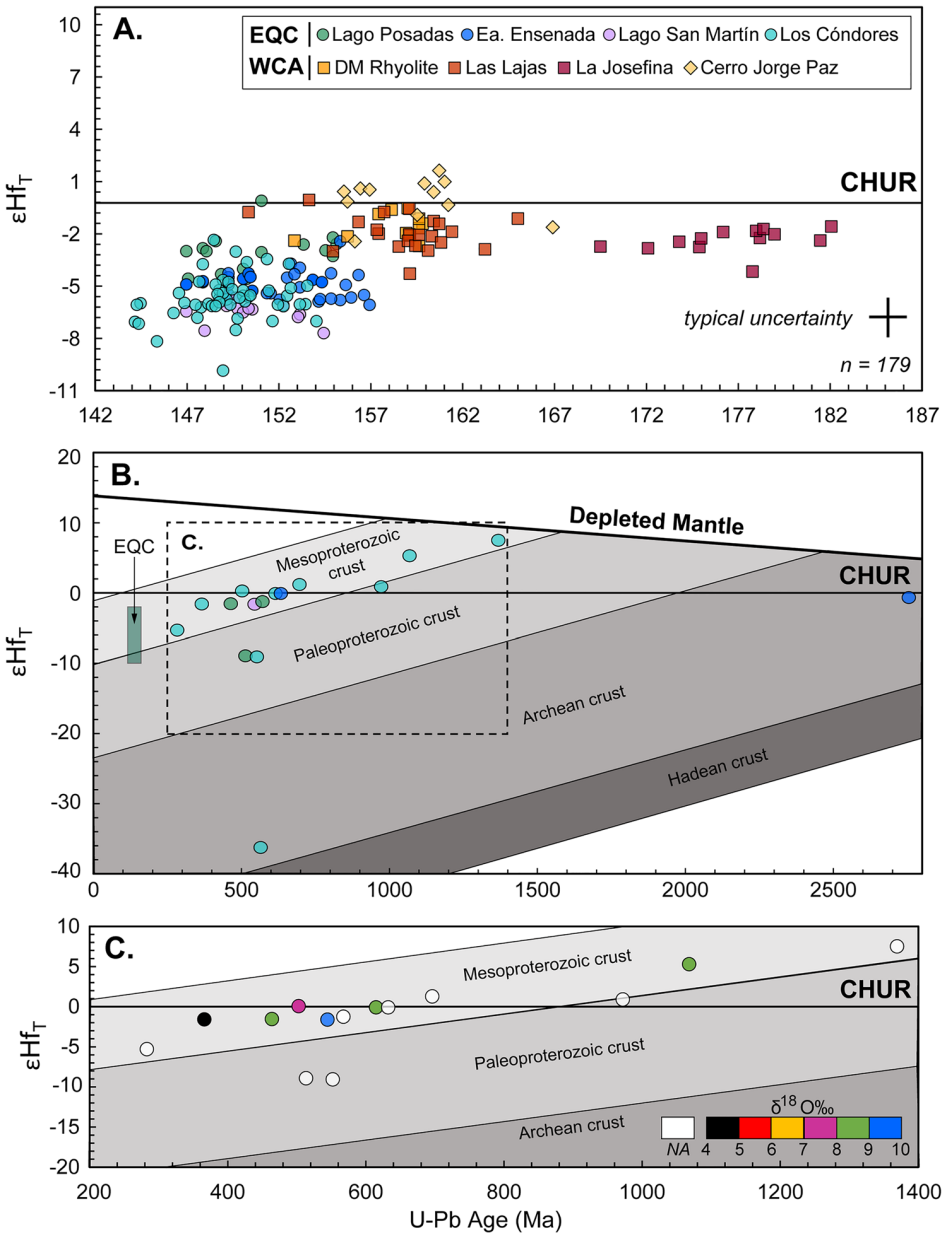
LA-ICP-MS εHf_T_ versus U–Pb age from the same zircon domain. **A** Most values for the EQC and WCA are below the Chondrite Uniform Reservoir (CHUR = 0), except for the CJP ignimbrite. **B** The EQC autocrystic zircon and most of the inherited cores follow an evolution along a similar slope, suggesting a magmatic source derived from melting of material with a Mesoproterozoic age. Colors of analyses correspond to the EQC locality where the core was analyzed. **C** Some inherited cores have elevated δ^18^O values that indicate periods of crustal reworking, and therefore, the εHf_T_ value represents a mixed signal

**Fig. 7:**
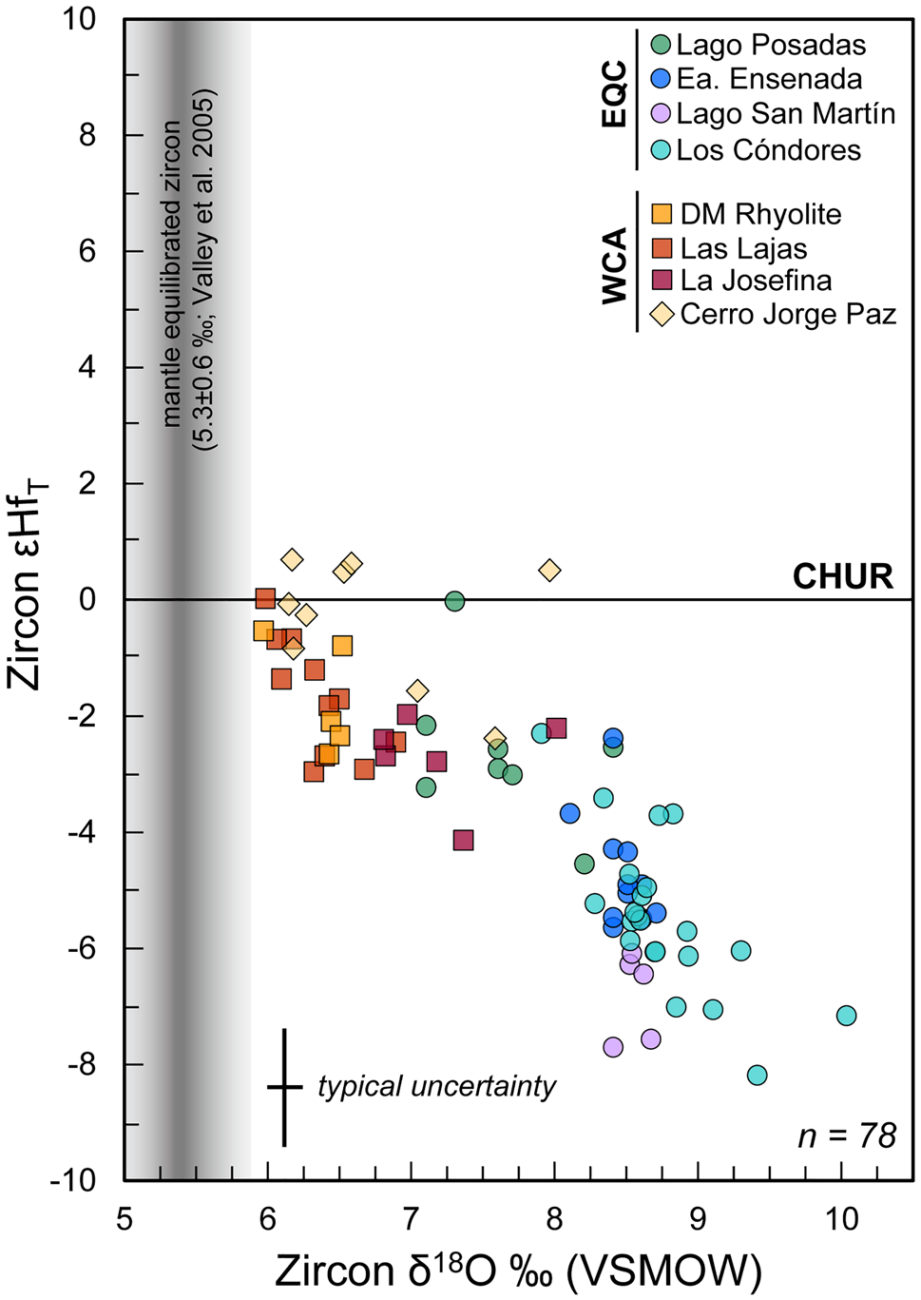
EQC and WCA Formations εHf_T_ versus δ^18^O zircon values. Samples from the EQC and WCA both display a semi-linear trend relative to the axes. Zircon compositions in equilibrium with mantle δ^18^O values (5.3 ± 0.6‰; Valley et al. 2005) are indicated by the shaded gray area. Although the EQC and CA zircon exhibit distinct ranges of εHf_T_ versus δ^18^O, all CASP zircon have oxygen values exceeding those of the mantle composition and most are below CHUR, where εHf_T_ = 0

Initial hafnium isotopic values in the EQC range from − 2.2 to − 8.1 εHf, excluding two outliers of 0.0 and − 9.8 (Fig. 6A). The negative hafnium isotope values of − 5.0 to − 8.1 correspond to the three EQC localities with the highest zircon oxygen isotope compositions (7.9–10.0‰) (Fig. 7). The zircon of the Lago Posadas ignimbrites with initial hafnium values of − 2.0 to − 4.5 correspond to the lowest δ^18^O, relatively, with values falling between 7.1 and 8.4‰.

#### Jurassic autocrystic zircon from the WCA

The hafnium and oxygen isotope compositions of zircon from the WCA show less variability per sample, with most analysis falling in between 6 and 7‰ δ^18^O (Fig. 5B) with the corresponding εHf values of + 1 to − 3.0 (Fig. 6A). The La Josefina rhyolite has an average hafnium isotopic composition of -2.3 (-1.5 to -4.1) and oxygen isotopic values ranging from 7 to 8‰ (Figs. 6 and 7). The Cerro Jorge Paz ignimbrite (DMMF3) ranges in hafnium composition from + 0.7 to − 2.4 (Fig. 6A), although it is similar in its oxygen isotope compositions with the DM rhyolite and Las Lajas ignimbrite with δ^18^O values of 6.2–8.0‰. Core to rim compositional variations are only observed in the Cerro Jorge Paz ignimbrite, with three core analyses of 7.5–8.0‰, overlapping with the compositions of the La Josefina rhyolite (Fig. 5B), and the corresponding rim compositions of 6.2 and 7.0‰ (Fig. 3). A single zircon in the Cerro Jorge Paz ignimbrite shows decoupling of the δ^18^O–Hf isotopic compositions (Fig. 3), where the rim has a lower oxygen isotope value of 6.2‰ with a negative εHf value of − 0.9, while the core has a higher value of 8.0‰ with an εHf of + 0.6.

#### Inherited zircon

Many of the inherited cores have an oxygen isotope composition similar to the values in the EQC zircon, or higher. In the Los Cóndores samples, most of the inherited cores are lower in δ^18^O ranging from 4.5 to 7.5‰ (Fig. 4; Table 2). Initial hafnium values in all samples also have a large range for the measured cores, ranging from + 1.2 to − 36.2. (Table 2). Plotting εHf_T_ through time (Fig. 6B) shows an evolution toward an increase in crustal-derived εHf values (i.e., decreasing εHf values) from the oldest cores of Grenvillian (1368 Ma, + 7.5 εHf) to Permian ages (283 Ma, − 5.1 εHf), with most of the inherited cores evolving along a similar slope.

**Table 2:** Inherited zircon dates with corresponding δ^18^O and/or εHfT value. Analyses are ordered sequentially, based on an origin from a Mesoproterozoic, Paleoproterozoic, or Archean crustal source as displayed in Fig. 6b. The few xenocrystic zircon corresponding to discordant age analyses (*n* = 5) are indicated with italic font

Sample	Sample ID (mount_zircon grain)	U–Pb (Ma)	± 2 S.D	δ^18^O ‰ (VSMOW)	εHf_T_ (CHUR)	T_DM_ (CHUR)
LCMF1	Z15_Zr6	185	2		− 4.9	1014
LCMF14	Z23_Zr9	196	6	7.5		
EEMF9	Z1_Zr6	231	10	9.2		
LCMF14	Z23_Zr4	240	6	5.8		
LCMF1	Z15_Zr6	283	4		− 5.1	1084
LCMF14	Z23_Zr10	366	8	4.5	− 1.6	1040
LCMF4	Z23_Zr1	357	6	9.5		
LPMF1	Z1_Zr11	464	5	8.1	− 1.5	1156
LCMF1	Z23_Zr12	503	12	7.3	+ 0.3	1070
LSMMF1	Z21_Zr26	544	6	9.2	− 1.6	1195
LPMF10	Z24_Zr24	567	5		− 1.3	1174
LCMF14	Z23_Zr24	615	8	8.5	− 0.1	1192
EEMF4	Z14_Zr8	634			0	1174
LCMF12	Z15_Zr14	696	12		+ 1.2	1178
LCMF14	Z23_Zr11	880	16	5.5		
LCMF14	Z23_Zr14	1068	144	8.9	+ 5.3	1347
LCMF4	Z23_Zr19	1368	43		+ 7.5	1517
LPMF1	Z24_Zr21	513	4		− 8.9	1452
LCMF14	Z15_Zr9	552	6		− 9.1	1434
LCMF14	Z15_Zr18	972	42		+ 0.9	1440
LCMF12	Z15_Zr5	565	8		− 36.2	2589
EEMF4	Z14_Zr16	2756	8		− 0.7	3045

Dates younger than 900 Ma are reported with their ^206^Pb/^238^U age, while dates older than 900 Ma are reported with the ^207^Pb/^206^Pb age

Oxygen isotope values are not systematically correlated with its U-Pb date in the same crystal, with high values (> 8.5‰) measured in both young (231 ± 10 Ma) and old (1068 ± 144 Ma) inherited cores (Fig. 6C). Many of the analyzed cores display high δ^18^O values (> 7‰) through time, suggesting a long-lived history of crust-derived magmatic sources.

### Zircon trace elements

**Fig. 8:**
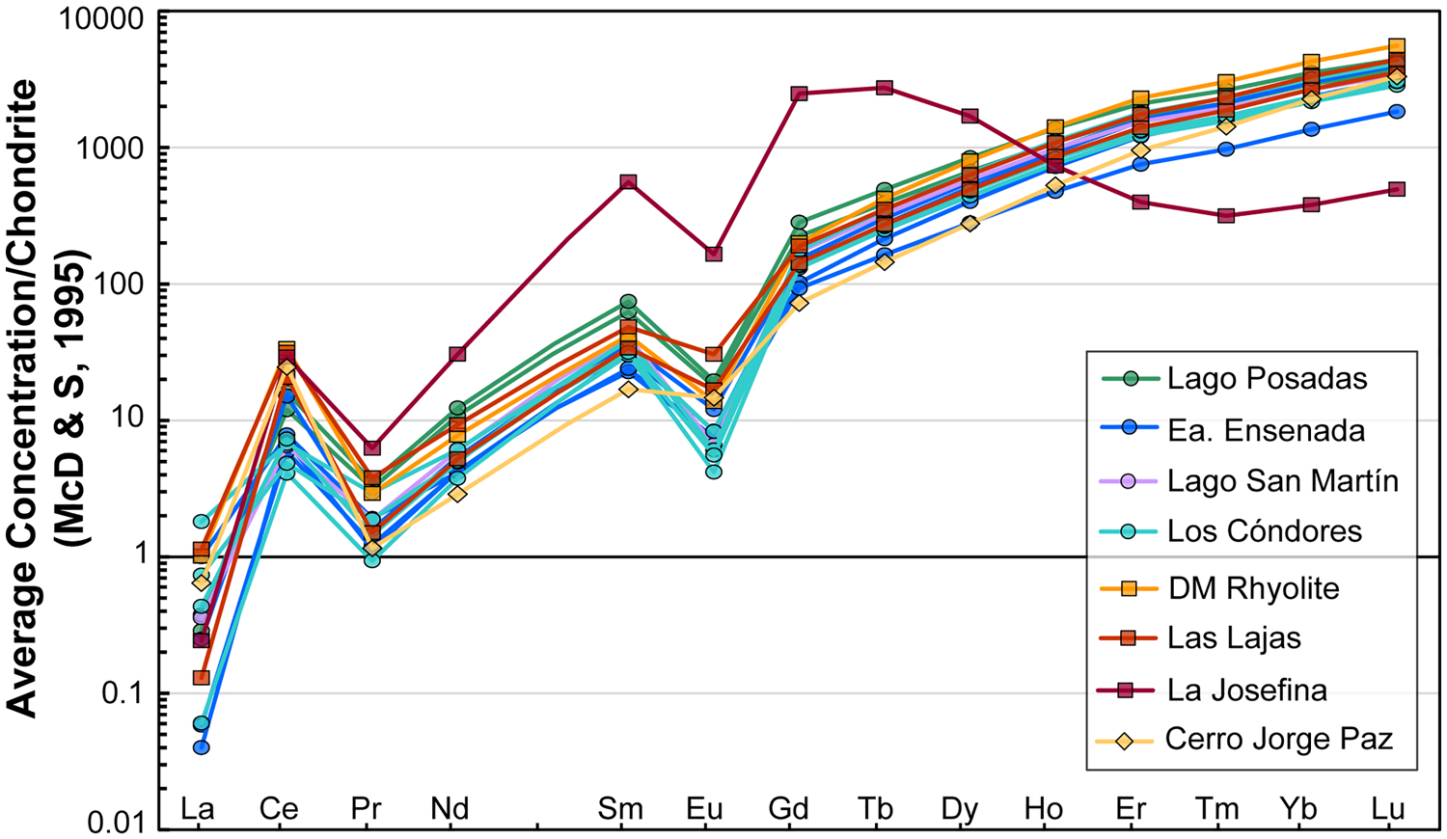
REE concentrations of EQC and WCA zircon. WCA zircon have similar REE to the EQC, except for the La Josefina rhyolite which displays a strong depletion in HREE, suggesting the presence of garnet in the melt source

Chondrite-normalized rare earth element (REE) trends in the EQC and WCA zircon show typical patterns of increasing concentration from light to heavy REE and have positive Ce and negative Eu anomalies (Fig. 8). There are no major differences in these trends between the two formations, though the WCA units have overall slightly higher REE concentrations than those of the EQC (Supplementary T10). An exception is the La Josefina rhyolite. Although it similarly has a REE pattern showing a positive Ce and a negative Eu anomaly, it also shows a steady decrease in heavy REE (Fig. 8). This is consistent with the bulk-rock REE pattern for LMJMF3, where a depletion in heavy REE is also observed (Foley et al. 2023).

**Fig. 9:**
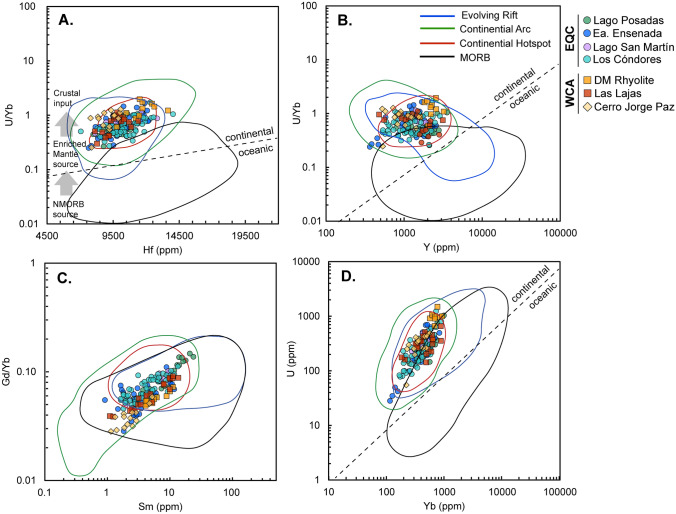
Zircon trace elements of (**A**) Hf (ppm) versus U/Yb, (**B**) Y (ppm) versus U/Yb, (**C**) Sm (ppm) versus Gd/Yb, and (**D**) Yb (ppm) versus U (ppm) are based on those designated by Grimes et al. (2015) to discriminate between tectono-magmatic provenance. The four fields of zircon compositions corresponding to unique tectonic regimes were drawn based on the datasets complied by Carley et al. (2014). Zircon compositions for both the EQC and WCA closely overlap and most closely follow the fields for continental hotspot zircon, but also extend into the field of continental arc zircon. Trace element concentrations for the La Josefina rhyolite (LMJMF3) are shown in Supplemental F4

To help us discern potential melting sources at depth, we use the discrimination diagrams of Grimes et al. (2015) and compare the EQC and WCA to zircon compositions that derive from known tectono-magmatic settings (Fig. 9). We outline the compositional fields for zircon that crystallized from melts originating in evolving rifts, continental arcs, continental hotspots, or MORB (mid-ocean ridge basalts) using the compilation dataset presented in Carley et al. (2014). Trace element plots show similar patterns between the EQC and WCA volcanic rocks. Zircon trace element compositions plot tightly for most samples in the continental hotspot field, based on zircon data from the Yellowstone caldera (Stelten et al. 2013); however, there is also a strong overlap with the continental arc field (Fig. 9). To better visualize the trends in trace element data, we excluded the La Josefina rhyolite from Fig. 9, due to the extremely high U concentrations (420–15,500 ppm) and depleted Yb concentrations (10–20 ppm), resulting in U/Yb values of 425–1190. Additional plots are available in Supplementary F4.

### Magmatic temperature constraints

**Fig. 10:**
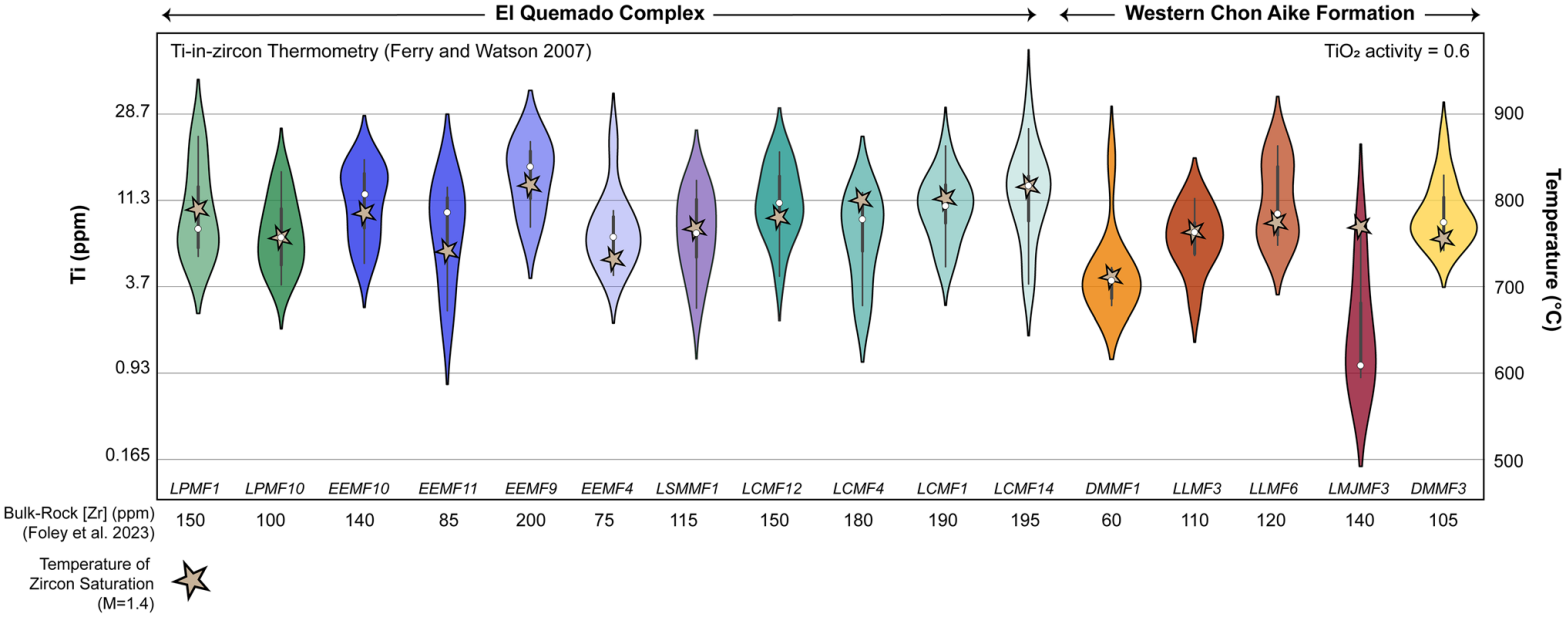
Violin plot of Ti-in-zircon temperatures based on the calibration of Ferry and Watson (2007). Concentrations of Ti (ppm) are plotted against calculated temperature (°C) on the y-axis. All Ti-in-zircon temperatures are calculated for TiO_2_ activities of 0.6. Samples which have average crystallization temperature exceeding the bulk zircon saturation temperature likely require a higher TiO_2_ activity than modeled (i.e., an αTiO2 of 0.8 =  ~ − 30 °C). Temperatures are similar across all ignimbrite samples with median values between 750 and 800 °C. The two WCA rhyolite domes (DMMF1 and LMJMF3) are lower in median temperature, though the extremely low temperatures of the La Josefina rhyolite likely do not reflect magmatic conditions

We estimate the temperature of zircon saturation using the thermometer of Watson and Harrison (1983). We assume that Zr behaved as an immobile element during post-eruption hydrothermal alteration and that alteration was not strong enough to change the overall mass significantly. The temperatures are calculated for each sample using the measured bulk-rock Zr concentration (ppm). The application of the zircon saturation equation depends on knowledge of the coefficient M = [Na + K + 2*Ca)/(Al*Si)], which was found to be a good proxy for the melt composition. Due to post-emplacement hydrothermal alteration, the bulk-rock samples have M values that range from 0.6 to 2.0 (Foley et al. 2023). A value of 1.4 was assumed based on the M value calculated for the least altered Las Lajas ignimbrite samples. This is in good agreement with typical values obtained for rhyolites, though differences in the estimated value for M result in relatively small errors in the calculated temperature (e.g., Miller et al. 2003). Zircon saturation temperatures for the EQC range from 730 to 820 °C (average 785 °C), while the WCA samples range from 710 to 780 °C with a lower average of 760 °C (Fig. 10).

Measurements of in situ zircon titanium concentrations and the Ti-in-zircon thermometer (Ferry and Watson 2007) were used to calculate the temperature of the melt at the time of zircon crystallization. We assume that the activity (α) of TiO_2_ is restricted between ~ 0.6 and 1 (Watson and Harrison 2005) and that αSiO_2_ = 1 given the evolved nature of the silica-rich magmas and large size of the quartz phenocrysts (> 1–3 mm; Foley et al. 2023). In our case, rutile saturation cannot be assumed at the time of zircon crystallization; thus, calculated temperatures are subject to uncertainty related to αTiO_2_. Average zircon Ti contents ranged from 8 to 16 ppm for the EQC (total individual analyses variations are 3 to 32 ppm) and 5 to 12 ppm (total individual analyses variations are 3 to 22 ppm) for the WCA. These average Ti contents translate into temperatures ranging from 775 to 850 °C and 730 to 815 °C (Fig. 10), respectively, for αTiO_2_ = 0.6. Assuming a higher αTiO_2_ of 0.8 would result in a decrease of ~ 30 °C.

### Oxygen isotope values of zircon–quartz pairs

**Fig. 11:**
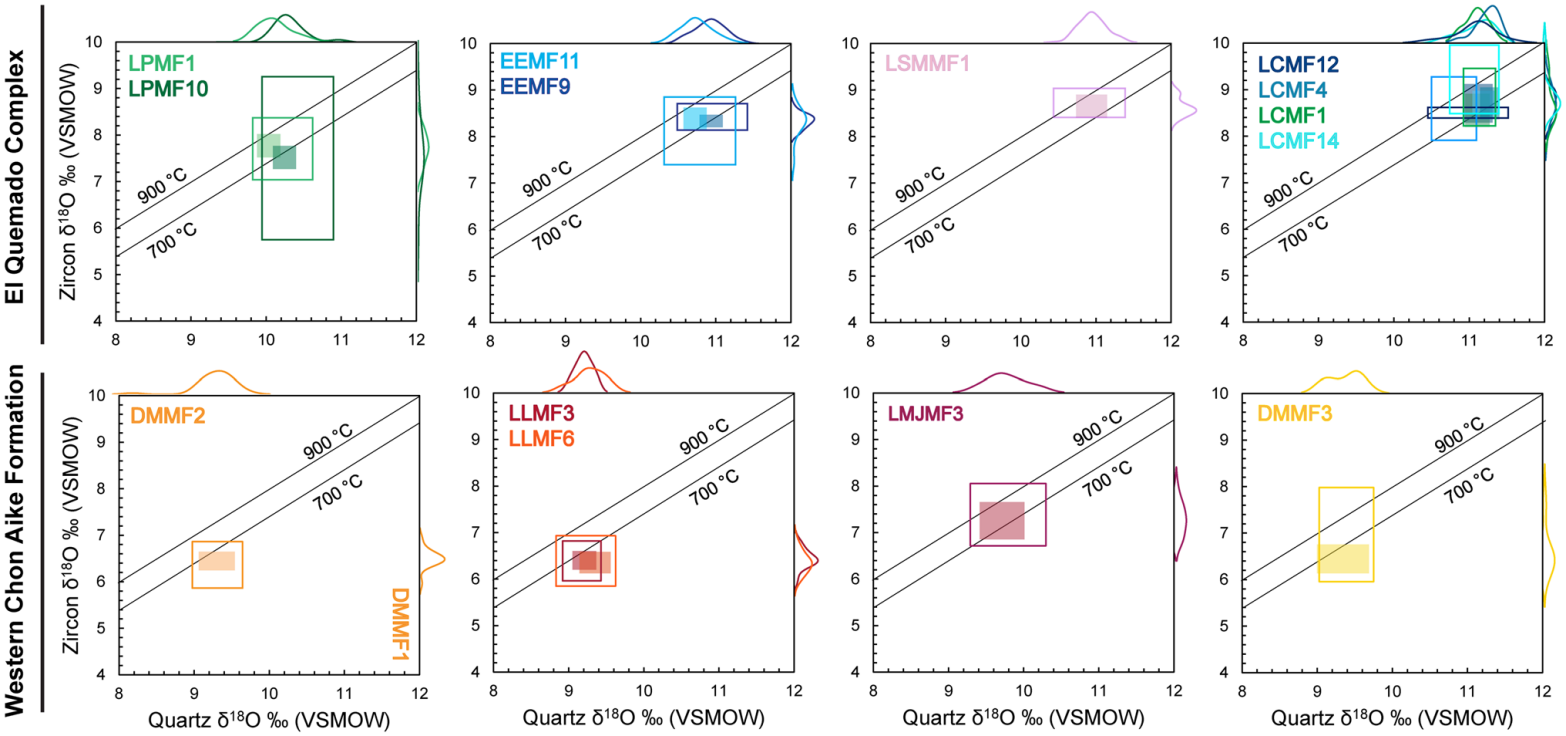
Oxygen isotope equilibrium fractionation diagrams displaying quartz and zircon equilibrium lines calculated for 700 and 900 °C. Quartz values from Foley et al. (2023) are plotted against zircon values from the corresponding sample. The total range of values (not including errors) are indicated by open boxes, while shaded areas highlight the largest proportion of analysis values (indicated by the KDE peak). Most quartz–zircon pairs for the same sample indicate isotopic disequilibrium. This suggests either differences in timing of saturation during evolution of the melt or coeval evolution of mineral phases in discrete melt pockets, prior to eruption. See text for discussion

We combine the detailed in situ oxygen isotope datasets for zircon and quartz phenocrysts extracted from the same bulk-rock sample (see Foley et al. 2023 for quartz SIMS δ^18^O values). To test for oxygen isotope equilibrium between quartz and zircon phenocrysts, their δ^18^O values should exhibit temperature-dependent fractionation with a fractionation factor (1000lnα ≈ δ^18^O_quartz_–δ^18^O_zircon_) between 2.4 and 3.3‰ at 900–700 °C, respectively (Zheng 1993). The isotope equilibrium lines for this temperature range along with the quartz–zircon paired measurements are shown in Fig. 11. Given that both mineral phases were extracted from bulk-rock separates, the original (i.e., magmatic) textural information was lost. Thus, without quartz–zircon pairs measured in direct contact, we plot the full range of δ^18^O values measured using kernel density estimates (KDE). The total range of δ^18^O values for each phase is illustrated by an open box and the region corresponding to the interquartile range of the δ^18^O measured values for each mineral is indicated by a shaded area.

The interquartile range of the δ^18^O values measured in EQC quartz and zircon plot largely between the equilibrium temperatures of 900 and 700 °C, with mean values closer to 800 °C. In comparison, the interquartile range for the quartz–zircon pairs from the WCA volcanic rocks (excluding LMJMF3) plot consistently in the lower temperature equilibration region, reaching below 700 °C. The La Josefina rhyolite exhibits an area with a similar range of δ^18^O quartz–zircon fractionation values to those of the EQC. Overall, the large range of δ^18^O values that plot outside the lines of equilibrium indicate that many zircon–quartz pairs are not in isotopic equilibrium for all 16 volcanic rocks considered.

## Discussion

### CASP U–Pb geochronology

The addition of 16 new samples dated by zircon U–Pb geochronology reveals that the EQC and WCA record distinct pulses of voluminous felsic volcanism in the CASP. The temporal progression of CASP magmatism documented by zircon U–Pb ages is overall consistent with a westward transition across Patagonia toward the paleo-Pacific margin (Pankhurst et al. 2000). However, the WCA and EQC remain unique in the CASP having a linear geometry of north–south coeval magmatism along > 200 km strike in the western Deseado Massif which progresses southwesterly until the cessation of the magmatic flare-up(s) marked by the eruptions of the EQC and Ibáñez Formations along the eastern Andean margin (Fig. 2).

The three WCA units dated within the western Deseado Massif closely overlap in age at ca. 159 Ma (Fig. 2). Only the rhyolite dome at the La Modesta locality is clearly older and unique in the western Deseado Massif at ~ 179 Ma, even when compared to the previously published U–Pb ages further north (ca. 156–159 Ma; Lopez 2006; Permuy-Vidal et al. 2021) and south (ca. 159–160 Ma; Dube et al. 2003; Moreira et al. 2009). This older age of 179 Ma overlaps with earlier phases of CASP volcanism within the North Patagonian Massif and in the eastern Deseado Massif (Navarrete et al. 2020; Matthews et al. 2021). The geochronology data of Matthews et al. (2021) for a succession of volcanic and subvolcanic felsic rocks reveal an extended history of magmatism in the eastern Deseado Massif that ranges from ca. 191 to 180 Ma and partially overlaps with the oldest CASP formations in the North Patagonian Massif. Collectively, ages of zircon U–Pb for the WCA indicate that magmatism progressed westerly across the Deseado Massif from ~ 191 to 160 Ma. The ages of magmatism in the western Deseado Massif are temporally distinct from those in the east, with an average zircon crystallization age of ca. 160 Ma (Fig. 2), which is why we choose to distinguish between western and eastern domains of the Chon Aike Formation.

The youngest felsic volcanism in the CASP is marked by the eruptions of the EQC that have a very narrow range of crystallization ages from 148 to 153 Ma. These zircon crystallization ages from the EQC ignimbrites document that there is no temporal trend from north to south along the eastern Andean Cordillera margin (Fig. 2). The ages presented here lie within the larger age range reported by SHRIMP and LA-ICP-MS U–Pb ages in the literature for the EQC, ranging from 147 to 156 Ma for correlative ignimbrite units (Pankhurst et al. 2000; Malkowski et al. 2016; Foley et al. 2022).

### Identifying magma sources in southern Patagonia

Considering the overall prolonged ca. 45 Myr duration of magmatism in the CASP, the EQC and the WCA magmatism occurred relatively close in space and time at ca. 150 and 159 Ma, respectively. Yet, the two formations exhibit distinct ranges of zircon oxygen and Hf isotope values. The zircon from the felsic melts of the EQC have characteristically high δ^18^O values from 7 to 9.5‰ (Fig. 5) and non-radiogenic initial εHf values (− 2.3 to − 8.1; Fig. 6), in comparison with the WCA zircon that have δ^18^O values ranging from 6 to 8‰ and the corresponding εHf_T_ values of − 4.0 to + 1.5. Notably, all CASP zircon measured have oxygen isotope values higher than zircon values in equilibrium with the mantle or with a mafic crust (e.g., 5.3 ± 0.6‰; Valley et al. 1998, 2005; Valley 2003) and a non-radiogenic Hf composition. Combined, these zircon compositions require contribution from ^18^O-enriched and older, low-εHf non-radiogenic felsic crustal material.

**Fig. 12:**
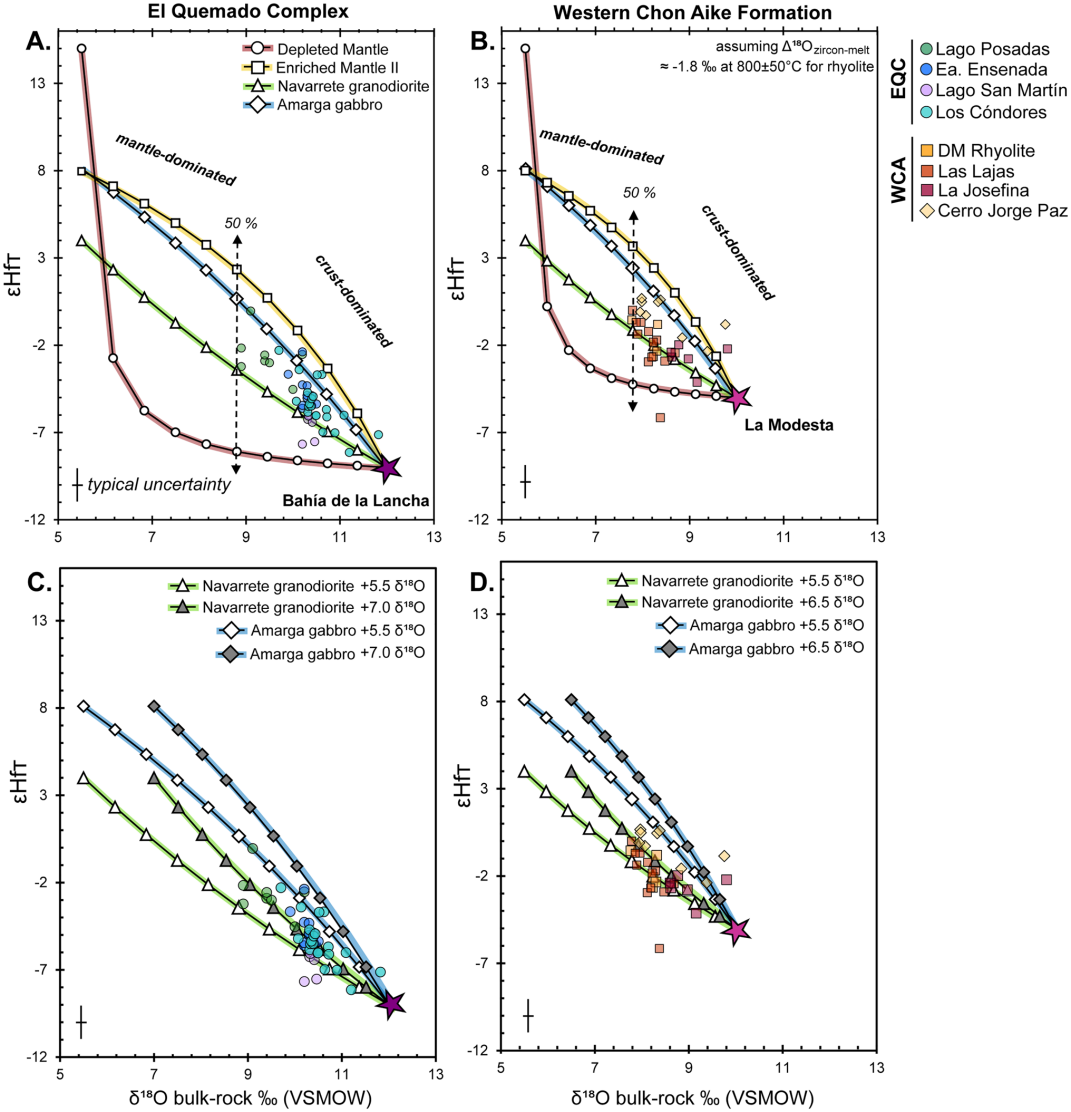
Binary mixing models of bulk-rock δ^18^O versus εHf_T_. Sample bulk-rock values are calculated from zircon δ^18^O assuming an average zircon-melt fractionation of − 1.8‰ at 800 ± 50 °C. **A** The EQC crustal endmember is modeled using the Bahía de la Lancha. **B** The WCA crustal endmember is modeled using the La Modesta Formation. Mantle endmembers are modeled with isotope values for the depleted mantle and enriched mantle II. Additional endmembers are constructed using published values in the Andean Cordillera with the Amarga gabbro (Torres del Paine) and the Navarrete granodiorite of the North Patagonian Massif. (**C**, **D**). An enriched mantle component with a δ^18^O composition of 7.0‰ best models the zircon compositions of the EQC, while the WCA is modeled with a slightly less enriched endmember of 6.5‰ δ^18^O. Even with an enriched mantle component, both the EQC and WCA represent crustal-dominated felsic magmatic systems with isotopic values that require > 50–80% of their melts to be derived from melting of respective crustal sources. See text for further discussion

To quantify the amount of crustal input, we calculated two-component mixing curves in δ^18^O-εHf space using calculated melt isotope compositions for the EQC and WCA (Fig. 12). The δ^18^O compositions of melt were computed from δ^18^O zircon values, using an average equilibrium temperature of 800 ± 50 °C for felsic rocks with a Δ^18^O_zircon-melt_ = − 1.8‰ (Bindeman et al. 2007). The EQC and WCA are both correlated to the region of southern Patagonia that is dominated by metasedimentary basement lithologies (Fig. 1), which are distinguished by their differences in maximum depositional ages. Although they are lithologically similar, Foley et al. (2023) showed that they differ in their bulk δ^18^O composition. Hence, we modeled each volcanic formation using the isotope compositions of each respective basement formation. For the EQC, we modeled the crustal endmember using the average bulk isotope value of the metasedimentary Bahía de la Lancha Formation (δ^18^O = 12‰; Foley et al. 2023). The εHf_T_ value is calculated using the equation of Vervoort et al. (2011) with the bulk-rock εNd_T_ value of − 6.6 measured in the same formation (Augustsson and Bahlburg 2003; Table 3). For the WCA, the crustal endmember is modeled using the La Modesta Formation with a bulk-rock δ^18^O value of 10‰ (Foley et al. 2023). Without published radiogenic isotope values for basement lithologies in the Deseado Massif, we estimated the εHf_160_ value to be − 5.0 using the average εNd_T_ value measured in the andesitic Bajo Pobre Formation, assuming this reflects the most juvenile value derived from partial melting of the crustal precursor (εNd_T_ range from − 3.8 to − 4.2; Pankhurst and Rapela 1995).

**Table 3:** Endmember mixing parameters

endmember	Hf (ppm)	O (ppm)	εHf_T_	δ^18^O_WR_ ‰
Depleted mantle (DM)	0.157^a^	450,000	+ 15.0^b^	+ 5.5^c^
Enriched mantle II (EMII_150_)	8.0^d^	450,000	+ 5.5^b^	+ 6.0^e^
Navarrete granodiorite	3.0^f^	450,000	+ 4.0^g^	varied
Amarga gabbro	5.2^h^	450,000	+ 8.1^i^	varied
Bahía de la Lancha Formation	4.0^j^	466,000	− 9.0^k^	+ 12.0^j^
La Modesta Formation	4.0^j^	466,000	− 5.0^l^	+ 10.0^j^

^a^Workman and Hart (2005)

^b^Zindler and Hart (1986)

^c^Mattey et al. (1994)

^d^Willbold and Stracke (2006)

^e^Eiler (2001)

^f^Pankhurst et al. (2006)

^g^Castillo et al. (2017)

^h^Müntener et al. (2018)

^i^Ewing et al. (2018)

^j^Foley et al. (2023)

^k^Augustsson and Bahlburg (2003)

^l^Pankhurst and Rapela (1995)

The geometry of the δ^18^O–Hf isotope array places constraints on the mixing process since the curvature is controlled predominately by the relative abundance of Hf in the endmembers. For both the EQC and WCA volcanic rocks, the isotope values cannot be reproduced via endmember mixing of the respective crustal source with either a depleted mantle or an enriched mantle source (Fig. 12A, B). Due to the concentrations of hafnium in either of the two mantle domains (e.g., DM = 0.157 ppm Hf, Workman and Hart 2005; EM-II = 8 ppm Hf; Willbold and Stracke 2006), both models result in a distinct curvature not seen in our data (Fig. 12 A, B). Instead, the near linear trends observed in both the EQC and WCA data (Fig. 12) require Hf concentrations in the mantle endmember which are closer to the crustal source (~ 3–5 ppm). Such mantle sources could be like those that produced the Hf isotopic compositions in zircon from the Amarga gabbro (ca. 29–30 Ma; Müntener et al. 2018) in the Torres del Paine Intrusive Complex (εHf_T_ =  + 8.1; Ewing et al. 2018) and the Navarrete granodiorite (ca. 281 Ma; Pankhurst et al. 2006) in the North Patagonian Massif (εHf_T_ =  + 4.0; Castillo et al. 2017). Without actual corresponding oxygen isotope data for these two model endmembers, we make assumptions on their δ^18^O composition and consider the effect of an enriched oxygen isotope composition (Fig. 12C, D). Given that these mantle-derived compositions have altered Hf isotope compositions due to subduction-related contamination of the underlying subcontinental lithospheric mantle, it is likely that their oxygen isotope compositions are similarly enriched relative to the depleted mantle value of 5.5 ± 0.2‰ (Mattey et al. 1994; Eiler 2001). We base our model endmembers using enriched oxygen values measured in arc systems (e.g., > 6‰ up to 7‰ δ^18^O; Bindeman et al. 2005; Dallai et al. 2019; González-Maurel et al. 2020; Cornet et al. 2022).

The covariant δ^18^O–Hf zircon arrays in Fig. 12 illustrate the interaction between two endmember components: (1) a modified (i.e., enriched) subcontinental lithospheric mantle and (2) metasedimentary basement lithologies. Based on the trajectories of the isotope arrays between the EQC and WCA, the mantle-derived material sourced from the subcontinental lithospheric mantle is modified beneath both the Andean Cordillera and the western Deseado Massif regions. The EQC values lie between the isotope values of the Amarga gabbro and the Navarrete granodiorite, with an enriched oxygen endmember (δ^18^O = 7.0‰) of the Navarrete granodiorite reproducing much of the EQC values (Fig. 12C). In the Deseado Massif, the WCA values are similarly modeled using the Navarrete granodiorite, though requiring a slightly less enriched oxygen isotope source (δ^18^O = 6.5‰; Fig. 12D). Given the difference in age to the Jurassic rhyolites, their relatively small volumes, and their distances to the EQC and WCA volcanic rocks, they are not the source region. Nevertheless, the Amarga gabbro and the Navarrete granodiorite document the presence of modified and enriched mantle compositions in the area that have persisted at least since the Permian and continued until the Paleogene in Patagonia. These mixing models are consistent with magmatic sources modeled for the genesis of the Permian granitoids in the North Patagonian Massif and in Tierra del Fuego (e.g., Castillo et al. 2017) and suggest commonality in the subcontinental lithospheric mantle compositions along the eastern half of Patagonia. The presence of multiple, unique mantle domains within Patagonia is supported by geochemical and isotopic studies of mantle xenoliths (e.g., Mundl et al. 2015; Schilling et al. 2017) and is discussed further in the following section (see Constraints on the crustal evolution of southern Patagonia).

Even with an enriched mantle source, the EQC and WCA are crust-dominated systems derived by melting of lithologies of similar composition (e.g., Foley et al. 2023). This is further supported by the consistencies in zircon trace element geochemistry between the two volcanic formations (Fig. 9). Hence, we interpret that the differences between the EQC and WCA zircon isotope values primarily reflect changes in the isotope composition of the basement source between the eastern Andean region and the western Deseado Massif, in addition to slight differences in relative proportions of endmember mixing where the WCA requires as much as 60% of a melted metasedimentary crust while EQC is closer to 70% on average (Fig. 12). The high δ^18^O values in the EQC (autocrystic zircon values of 7–9.5‰) reflect a crustal source region with similarly high δ^18^O values. In comparison, although the WCA zircon δ^18^O values are not as enriched as the EQC (Fig. 5), they nevertheless represent a crust-dominated system (Fig. 12). Thus, tracing the origin of high-δ^18^O magmas is demonstrated in this difference between the EQC and WCA: producing a magma with resulting high δ^18^O value requires both a source with an elevated bulk-rock δ^18^O (> 10‰) composition and significant mass contribution (> 50–90%) during partial melting.

### Magma genesis

The two-component zircon mixing models for the EQC and WCA indicate a dominant felsic crustal component in the magmatic source (e.g., > 50–80% by mass; Fig. 12). To generate the large volumes of felsic magmas observed (ca. 10,000 km^3^ and 20,000 km^3^ for the EQC and WCA, respectively; Pankhurst et al. 1998), a petrogenetic model that satisfies the following observations is required: (1) widespread and voluminous production of felsic magmas with isotope compositions that suggest open-system mixing between crustal and enriched mantle sources, and (2) effective mixing and homogenization of magma batches across the affected region, prior to the rapid eruption of explosive material suggested by U–Pb zircon crystallization ages within the EQC and WCA, which are often within error of the method.

Hybrid magmas are difficult to achieve thermally within the shallow, cold crust, as the heat available for crustal melting and/or assimilation is limited by the volume of injected mafic magmas (e.g., Annen and Sparks 2002; Annen et al. 2006). Therefore, our observations for the EQC and WCA are more consistent with the generation of felsic magmas primarily within a hot crustal environment found in either the lower crust or at a relatively shallow depth in an extensional environment. Based on phase petrology, Foley et al. (2023) suggested that anatexis is most efficient at lower pressures (~ 5 kbar). Combining these arguments, we suggest that crustal melting occurred at the base of an extended, thinned crust. Rapid extension would provide the hot ambient temperatures necessary for significant crustal melting and mixing. The δ^18^O-εHf mixing models suggest open-system mixing with mantle melts (Fig. 12), whereby magmas were modified in crustal MASH zones (mixing, assimilation, storage, and homogenization; Hildreth and Moorbath 1988), with heat primarily provided in the form of basaltic underplating (e.g., Bergantz 1989; Dufek and Bergantz 2005). Efficient mixing between distinct melt sources in the thinned lower crust led to the development of intermediate to felsic magmas with a hybridized and uniform isotopic signature (Riley et al. 2001).

Interestingly, any trace of the history of open-system hybridization was not recorded in the zircon crystal record, as both the intra- and inter-sample isotopic records are homogeneous and with no difference in isotope values between zircon core and rim values (Figs. 3 and 4). Both the scale of isotope homogeneity (e.g., north to south through the EQC and west to east through the WCA) and the consistency of isotope values within different zircon populations for each sample suggest that zircon saturation occurred after homogenization. For most samples, zircon δ^18^O values have greater variation within a given sample population (9 out of 14 samples have a variance > 1.0‰; Fig. 5) than the corresponding phenocrystic quartz (all < 1.0‰). One explanation for the divergence in isotopic equilibrium could be that zircon records a longer history of magmatic evolution (i.e., saturation prior to quartz) and/or some of the zircon grains are antecrystic and recycled from an earlier phase of magmatism (e.g., Miller et al. 2007; Gaynor et al. 2023). However, based on petrographical observations, we determine that quartz was not a late-stage crystallizing phase. Quartz phenocrysts are often large (> 1–3 mm; Foley et al. 2023) and are similar in size to coexisting feldspar phenocrysts, thus supporting a long magmatic history. Additionally, neither quartz nor zircon δ^18^O values change through time, as both the lower and upper ignimbrites from an eruptive sequence in the EQC do not significantly change in composition (Fig. 11). Therefore, we conclude that the oxygen isotope disequilibrium between quartz and zircon more likely reflects crystallization in spatially discrete melt pockets within a single reservoir, such that each phase inherits the isotopic composition from only the immediate melt (e.g., Bindeman and Melnik 2016).

### Constraints on the crustal evolution of southern Patagonia

Given the high crustal proportions involved in magma genesis, isotopic measurements within the CASP volcanic rocks provide an indirect sampling of the crustal reservoirs of Patagonia and the Antarctic Peninsula. Broadly used, depleted mantle Hf model ages (T_DM_) provide constraints on the timing of melt extraction events (e.g., DePaolo et al. 1991). Here, we assume a linear evolution of Lu/Hf = 0.015 (Goodge and Vervoort 2006) for a newly extracted crust through time. To delineate some uncertainties involved in using zircon Hf model ages in comparison to bulk-rock model ages, we combine tracers of oxygen and Hf isotopes in zircon to provide a relative constraint on the measure of reworked older continental crust (Hf) along with the incorporation of supracrustal material (oxygen isotopes). Zircon with ^18^O-enriched isotope compositions reflect periods of crustal reworking, hence, the calculated T_DM_ age reflects mixing between a mantle-derived source (often assumed to be depleted mantle) and a crustal source (e.g., Dhuime et al. 2012; Cornet et al. 2022).

**Fig. 13:**
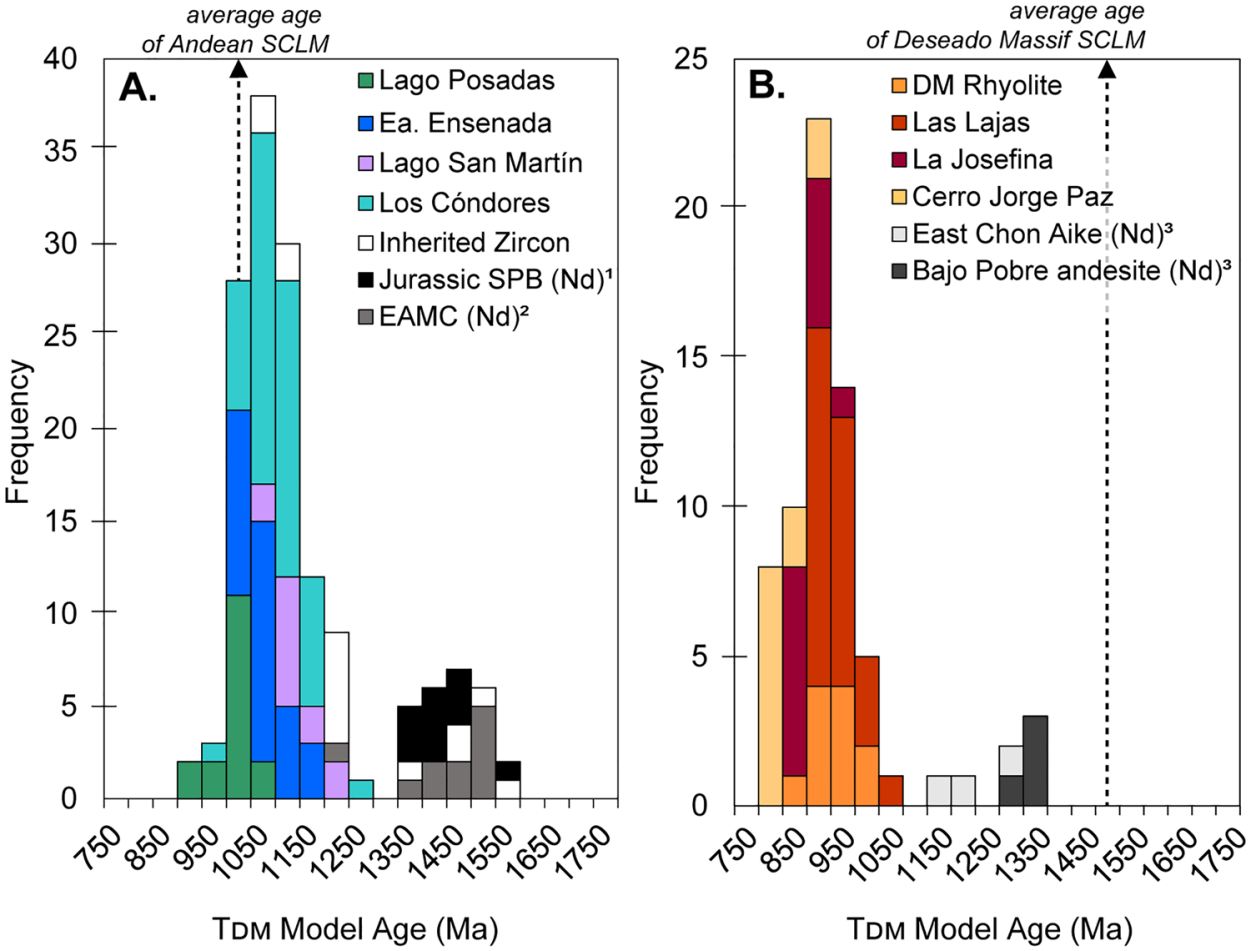
Comparison of depleted mantle model ages for lithologies associated with (**A**) the EQC within the southern Andes and (**B**) the WCA in the western Deseado Massif. CASP zircon Hf model ages are compared against published Nd data for the Southern Patagonian Batholith (1. Hervé et al. 2007), the Bahía de la Lancha (EAMC; 2. Augustsson and Bahlburg 2003), and additional CASP formations including the andesitic Bajo Pobre Formation and eastern Chon Aike Formation in the Deseado Massif (3. Pankhurst and Rapela 1995). In the Deseado Massif, the average age for the formation of the subcontinental lithospheric mantle (SCLM) is 1.5 Ga (Re–Os model ages; Mundl et al. 2016; Schilling et al. 2017), while xenolith model ages in localities closest to the eastern Andean margin are distinctly younger in age at ca. 1.0 Ga

In the Deseado Massif, the average age for the formation of the subcontinental lithospheric mantle is estimated to be 1.5 Ga, calculated using Re–Os isotopic data from mantle xenoliths (e.g., Mundl et al. 2015; Schilling et al. 2017), while model ages in the Andean region are considerably younger at ca. 1.0 Ga. Interestingly, bulk Neodymium model ages for the Bahía de la Lancha in the Andean region are older than the mantle xenolith model ages and are instead closer in age to those of the Deseado Massif at ca. 1.3–1.5 Ga (Fig. 13) and support their sediment origin from the active margin in the eastern Deseado Massif during the Paleozoic (Augustsson and Bahlburg 2003). Although the La Modesta Formation does not have complementary radiogenic values, it likely has radiogenic compositions close to those of Bahía de la Lancha Formation as they represent sediments derived from a similar source (Permuy-Vidal et al. 2014).

In the EQC, hafnium T_DM_ ages of > 1.0–1.2 Ga support derivation from recycling of the Paleozoic Bahía de la Lancha sediments in the lower crust (Fig. 13A). The younger ages of < 1.0 Ga at Lago Posadas are consistent with increasing input from mantle sources. In the Deseado Massif, the andesitic Bajo Pobre Formation, which is generated via partial melting of the lower crust and considered a parental magma for the CA Formation (e.g., Pankhurst and Rapela 1995), has Nd T_DM_ ages of ca. 1.3 Ga. Although this age for the Bajo Pobre Formation is consistent with the ages of the subcontinental lithospheric mantle formation, it is unclear if it represents the age of a lower crustal formation at this time, or the ages of its sedimentary provenance (i.e., La Modesta Formation). In comparison, Hf T_DM_ ages in the WCA are significantly younger (average 900 Ma) and reflect a greater proportion of mixing between crustal and mantle sources (Fig. 13B).

In addition to depleted mantle model ages, combining U–Pb with oxygen and hafnium isotope measurements in EQC inherited zircon provides valuable insights on the isotopic evolution of the crustal sources in southern Patagonia. If the Hf isotope composition of the crustal source material followed an inferred Lu–Hf crustal evolution path, zircon with similar model ages might originate from similar crustal domains with consistent T_DM_ ages of 1.0 to 1.5 Ga in zircon with the corresponding U–Pb ages ranging from Late Jurassic to Mesoproterozoic (Fig. 6B). The elevated oxygen isotope values in these older zircon grains also indicate that mixtures of older reworked materials with new (younger) mantle-derived material and the primordial continental crust from which they are sourced must be even older than is suggested by the calculation (e.g., > 1.1 Ga). Although the oldest, ca. 1.37 Ga, inherited zircon does not have a corresponding δ^18^O measurement, the T_DM_ age of 1.5 Ga is consistent with the average age of subcontinental lithospheric mantle in the Deseado Massif and supports the idea of lower crustal formation at this time.

### Tectonic framework considerations

**Fig. 14:**
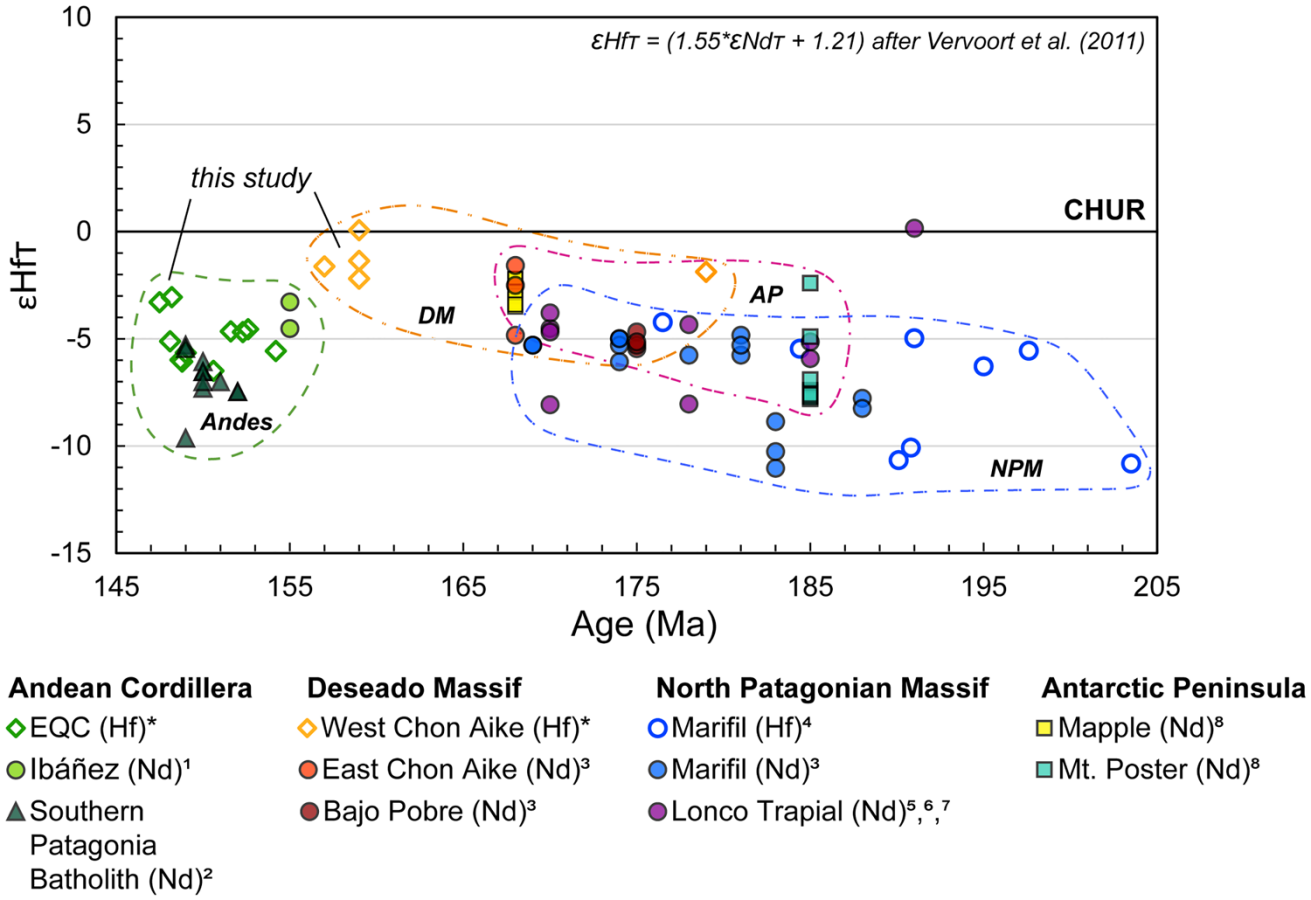
Comparison of radiogenic (Hf and Nd) isotope values across the CASP. Data are compiled from both bulk-rock (Nd) and in situ measurements (Hf in zircon). Dashed lines enclose formations which are differentiated by regions: Andean Cordillera, Deseado Massif, North Patagonian Massif, and the Antarctic Peninsula. All formations in the CASP, including both felsic and intermediate volcanic rocks, are below CHUR and support commonality in their crustal petrogenesis throughout the province. Epsilon Hf values are recalculated from εNd using the equation after Vervoort et al. (2011). Numbered references are as follows: 1) Parada et al. 1997; 2) Hervé et al. 2007; 3) Pankhurst and Rapela 1995; 4) Strazzere et al. 2021; 5) Zaffarana et al. 2020; 6) Dejonghe et al. 2002; 7) Bouhier et al. 2017; 8) Riley et al. 2001. The isotope data for the EQC and west Chon Aike Formations are from this study (denoted with an asterisk)

The isotope data of zircon presented here for the EQC and WCA are consistent with those of other formations in the CASP (Fig. 14). The broad consistency in both felsic and intermediate volcanic rocks (e.g., Lonco Trapial Formation) with a crustal isotopic signature supports a common petrogenesis for the CASP, as proposed before (e.g., Pankhurst and Rapela 1995; Pankhurst et al. 1998; Riley et al. 2001). We suggest that the main differences in isotope values through space and time likely reflect changes in contributing basement crustal source between the North Patagonia Massif, Deseado Massif, Andean region, and the Antarctic Peninsula (Fig. 1).

**Fig. 15:**
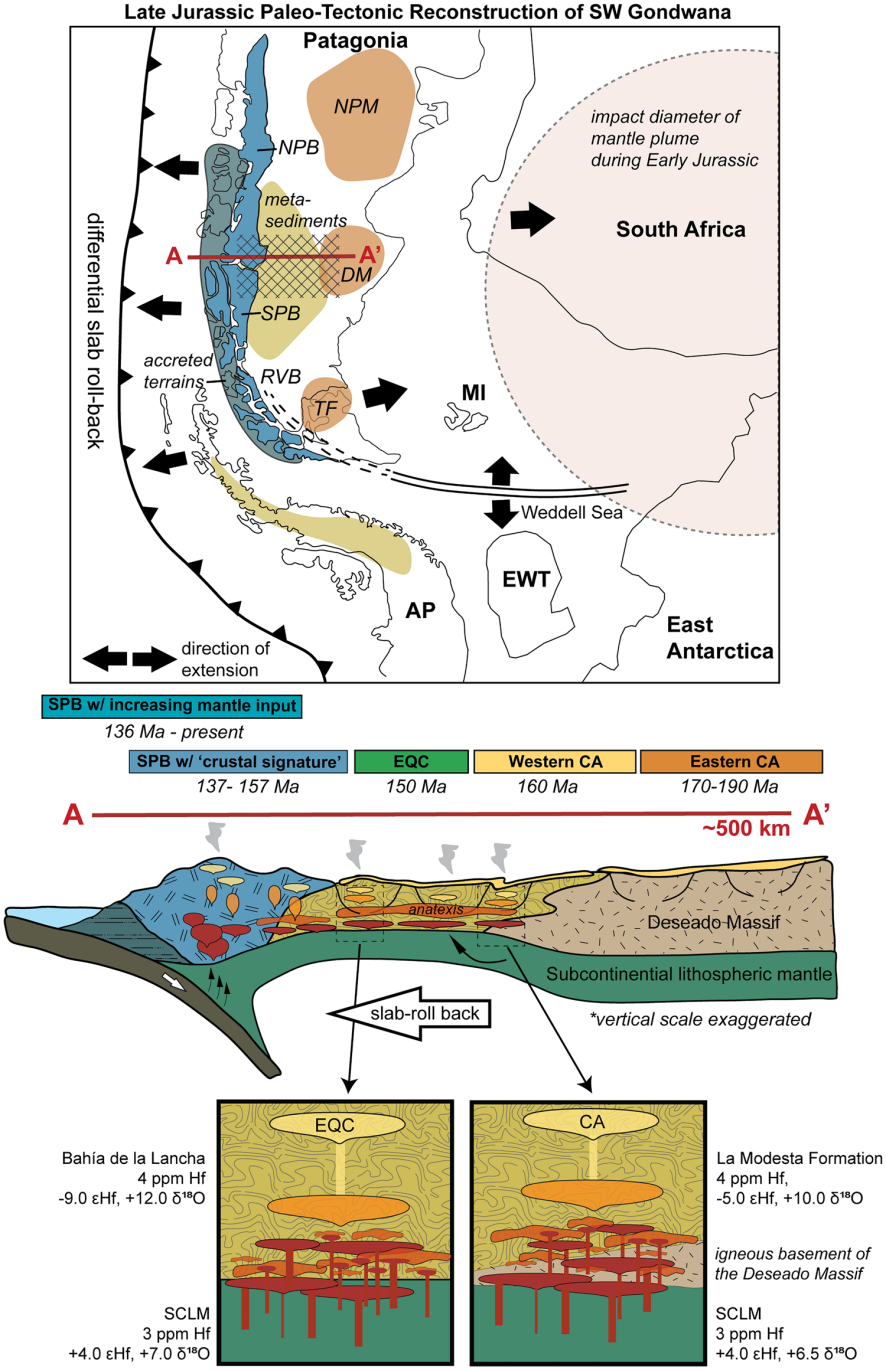
Paleotectonic reconstruction of southwest Gondwana during the Late Jurassic. Widespread crustal extension occurred following the differential slab rollback toward the western margin of the continent. Rifting associated with the opening of the Rocas Verdes back-arc basin (RVB) coincides with the Weddell Sea Formation during the Late Jurassic, suggesting the presence of a triple junction (Bastias et al. 2021). Direction of extensional forces (black arrows) are drawn from the reconstructions by Lovecchio et al. (2020). Cross-sectional illustration (A–A’ is ~ 500 km distance) of felsic magma generation in the lower crust by crustal anatexis and variable mixing with enriched mantle melts sourced from the subcontinental lithospheric domains. Although a 2D cross section is depicted, a larger region of southern Patagonia is affected coevally (crosshatch pattern in Patagonia). During the Cretaceous, the reposition of the subducting slab and commencement of normal subduction along the Pacific margin is interpreted based on the increase of depleted mantle isotope signatures of the Northern (NPB) and Southern Patagonian Batholith (SPB; Pankhurst et al. 1999; Hervé et al. 2007). Abbreviated crustal blocks: Ellsworth-Whitmore Terrain (EWT), Antarctic Peninsula (AP), and the Malvinas Islands

Since volcanism associated with magmas generated via crustal anatexis is considered rare (e.g., Moyen et al. 2021), largely due to the thermal constraints of crustal anatexis and/or processes of assimilation (e.g., Glazner 2007; Heinonen et al. 2021), the CASP requires an overlap of exceptional circumstances necessary to drive felsic volcanism of this scale and through time. Both the EQC and WCA felsic magmas were generated within the region of southern Patagonia dominated by felsic metasedimentary lithologies deposited in a paleo-arc environment (e.g., turbidite and shallow marine units; Fig. 15). Although we cannot completely rule out a contribution from additional crustal sources, the consistencies in Hf isotopic compositions of detrital zircon grains analyzed from the Bahía de la Lancha Formation and additional units within the Eastern Andean Metamorphic Complex, together with similar detrital age components (Augustsson et al. 2006; Suárez et al. 2019b) as those inherited zircon cores measured here, support the source for the EQC to be derived from partial melting of this metasedimentary lithology.

In comparison, exposures in the eastern Deseado Massif comprise an older Proterozoic crystalline basement. Due to extensive burial, largely from the Jurassic volcanic rocks and Mesozoic basalt flows, the nature of the connection between the igneous-metamorphic basement outcrops in the eastern Deseado Massif and the metasedimentary La Modesta Formation is not exposed. Therefore, we illustrate this boundary as a depositional contact (Fig. 15) that was later accreted to the crystalline basement during the Mid- to Late Paleozoic (e.g., Moreira et al. 2005, 2013). A contribution from this older Proterozoic basement could aid in explaining the geochemical and isotope variability exhibited in the CA zircon values. However, zircon trace element values in the WCA are similar to the EQC and are more consistent with an origin from sedimentary protoliths derived from a LIL-depleted upper continental crust source (Figs. 8 and 9).

CASP magmatism largely occurred during an extensional period that resulted in widespread crustal melting within a high heat flux environment (Pankhurst et al. 2000; Riley et al. 2001; Falco et al. 2022; Foley et al. 2023). The temporal and spatial overlap with the continental Karoo and Ferrar mafic large igneous provinces (ca. 182–183 Ma; Burgess et al. 2015; Greber et al. 2020; Muedi et al. 2022) led some authors to suggest that crustal melting occurred in response to extensive basaltic underplating, sourced from a nearby plume (e.g., Storey and Kyle 1997; Pankhurst et al. 2000; Storey et al. 2013). However, a significant mass input is not supported by radiogenic data (Fig. 14) from the temporally overlapping CASP formations in the North Patagonian Massif and in the eastern Deseado Massif (e.g., Pankhurst and Rapela 1995; Strazzere et al. 2021; Falco et al. 2022). Furthermore, recent modeling restricts the thermal influence of the mantle plume to a diameter of ca. 2000 km (e.g., Lovecchio et al. 2020), indicating that a plume model cannot account for the widespread felsic magmatism along the southwest Gondwanan margin (Fig. 15).

The southwest Gondwanan margin (Fig. 15) was likely pulled apart by plate boundary processes operating on either side of the supercontinent (Storey et al. 1992; Storey and Kyle 1997; Lovecchio et al. 2020 and references therein). Recent publications argue for the CASP magmatic flare-up(s) following differential rollback of the subducting oceanic plate that triggered continental dispersal during the Jurassic (e.g., Echaurren et al. 2017; Navarrete et al. 2019; Lovecchio et al. 2020; Bastias et al. 2021). Along the southern Andes, during the Late Jurassic, crustal extension formed both back-arc basins and rift zones adjacent to the evolving arc (e.g., Rocas Verdes Basin; Calderón et al. 2016; Muller et al. 2022).

The earliest evidence for active subduction in the Southern Patagonian Batholith is a gabbro dated at 157 ± 3 Ma (Hervé et al. 2007). However, geochemical and isotopic characteristics of the ca. 150 Ma granodiorites at latitudes parallel to the felsic volcanism of the EQC (49–50°S, Fig. 2) are consistent with their formation by crustal melting during regional extension (Hervé et al. 2007). Steepening of the subducted slab and trench-ward migration would have enhanced back-arc extension and lithospheric thinning (Echaurren et al. 2017). Preferential asthenospheric decompression melting due to lithospheric thinning increased the volume of the mantle input as underplated melts into the lower crust and provided the ideal conditions for widespread lower crustal anatexis.

Within this region of southern Patagonia, the juxtaposition of fertile, low-grade metasediments to the mantle from which mafic melts are extracted provide the necessary thermal environment for the widespread partial melting of the crust (Fig. 15). The pressure and temperature environment for the generation of the EQC and WCA felsic melts was identified by thermodynamic models for fluid-absent melting of the Bahía de la Lancha and La Modesta Formation graywacke lithologies at 900 °C and 5 kbar, which corresponds to a mid- to lower crustal depth of ca. 20 km within an extending crust (Foley et al. 2023). The absence of a garnet signature in the REE pattern restricts the depth of melting for both the EQC and WCA during the Late Jurassic, although the REE pattern in the La Josefina dome (Fig. 8) does indicate garnet in its crustal source during an earlier phase of volcanism in the western Deseado Massif at ca. 179 Ma.

## Conclusions

We discussed a large set of new zircon-based data from the Chon Aike Silicic Large Igneous Province that further highlights the significance of crustal anatexis for the formation of voluminous silicic magmas. Zircon from the EQC have ^18^O-enriched values (7–9.5 ‰) with correspondingly negative initial εHf (− 2.0 to − 8.0). Zircon from the WCA, in contrast, have less elevated δ^18^O values (6–7‰) and initial εHf values which trend toward increasing radiogenic Hf compositions (− 4.0 to + 1.5). Based on trace element concentrations in the La Josefina Dome, the oldest magmatism in the western Deseado Massif (ca. 179 Ma) likely resulted from crustal melting at a depth where garnet was stable, with the highest oxygen values (7–8‰) and initial εHf values of ca. − 3.0 measured in the WCA zircon. In comparison, the felsic volcanism at ca. 160 Ma is characterized by similarly elevated oxygen isotope values and a larger radiogenic Hf component. The EQC marks the final CASP magmatic flare-up at ca. 150 Ma along the eastern Andean Cordillera, following the differential rollback of the subducting oceanic plate toward the paleo-Pacific margin.

The isotope values are semi-linearly distributed between the endmember components of an enriched subcontinental lithospheric mantle and metasedimentary basement lithologies. Both the WCA and EQC felsic magmas represent crustal-dominated systems that require on average 60 to 70%, respectively, of their melts to be derived from the partial melting of a metasedimentary basement. Our observations for the EQC and WCA are consistent with the generation of the felsic magmas primarily within lower crustal MASH zones, where a significant volumetric contribution derives directly from partial melting of the crust as the primary source of the CASP melts. Despite a limited outcropping of mafic rocks in Patagonia, δ^18^O–Hf mixing models suggest open-system mixing with enriched mantle-derived melts.

Given the high crustal proportions involved in magma genesis, the combined oxygen and hafnium isotope measurements within the CASP provide an indirect sampling of the crustal reservoirs throughout Patagonia and the Antarctic Peninsula. Additionally, most of the Hf T_DM_ ages from inherited zircon indicate a Mesoproterozoic crust origin and have U–Pb dates reflecting the major Gondwanan orogenic cycles recorded in South America (e.g., Famatinian, Pampean, and Grenvillian; Hervé et al. 2003; Pankhurst et al. 2003; Ramos 2008). The oxygen isotope compositions of the inherited zircon cores are dominated by “supracrustal” elevated δ^18^O values, indicating that the periods of magmatism at the corresponding U–Pb date are dominated by geological processes which led to crustal reworking, as opposed to periods of crustal growth. Igneous zircon with high δ^18^O compositions are likewise documented along the western paleo-Gondwanan margin, including the Permian granitoids of Tierra del Fuego (e.g., Castillo et al. 2017) and the Permian–Triassic magmatic rocks of the Antarctic Peninsula (e.g., Bastias et al. 2019; Castillo et al. 2020). Sediments are the dominant reservoir of high δ^18^O material on Earth. The availability of sediments with high δ^18^O signatures and the subsequent magmatic recycling along the paleo-Pacific Gondwanan continental margin was significant for the generation of high δ^18^O magmas throughout the Paleozoic and Mesozoic.

### Supplementary Information

Below is the link to the electronic supplementary material.

Supplementary file1 (DOCX 4205 KB)

Supplementary file2 (XLSX 546 KB)

## Data Availability

All data obtained during this study are included in this published article and its supplementary files.
