# Supplementary material for: Identifying crustal contributions in the Patagonian Chon Aike Silicic Large Igneous Province
Source: Contrib Mineral Petrol. 2023 Oct 24;178(11):80. doi: 10.1007/s00410-023-02065-1 (PMC11008082; doi:10.1007/s00410-023-02065-1)

**Supplementary Figures**

**SF1.** Concordant LA-ICP-MS ^206^Pb/^238^U analyses for the volcanic units of the EQC and WCA. (A) EQC samples are arranged spatially from north to south and are arranged from top through bottom for an ignimbrite sequence at a single location. (B) Samples of the WCA are arranged spatially west to east. Individual analyses which are included in the average crystallization age calculation are colored by sample; analyses excluded in the mean weighted age are grey. The calculated MSWD is indicated below each crystallization age.

**
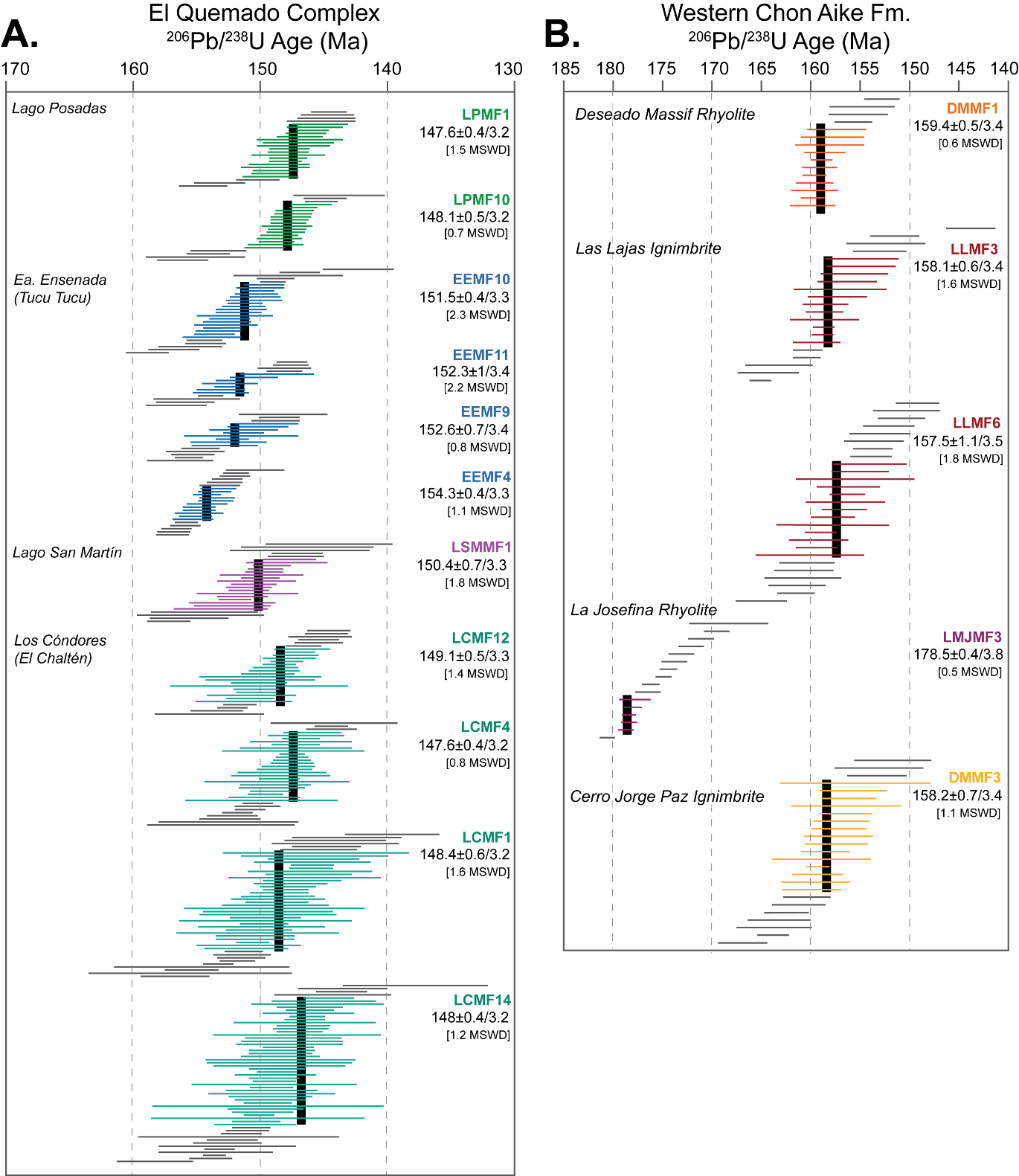
**

**SF2.** Concordia diagrams for the 16 CASP volcanic units which include primarily ignimbrites and minor rhyolitic flows. Two diagrams are displayed for each sample, including one for all analyses and the second including only concordant Jurassic aged analyses.


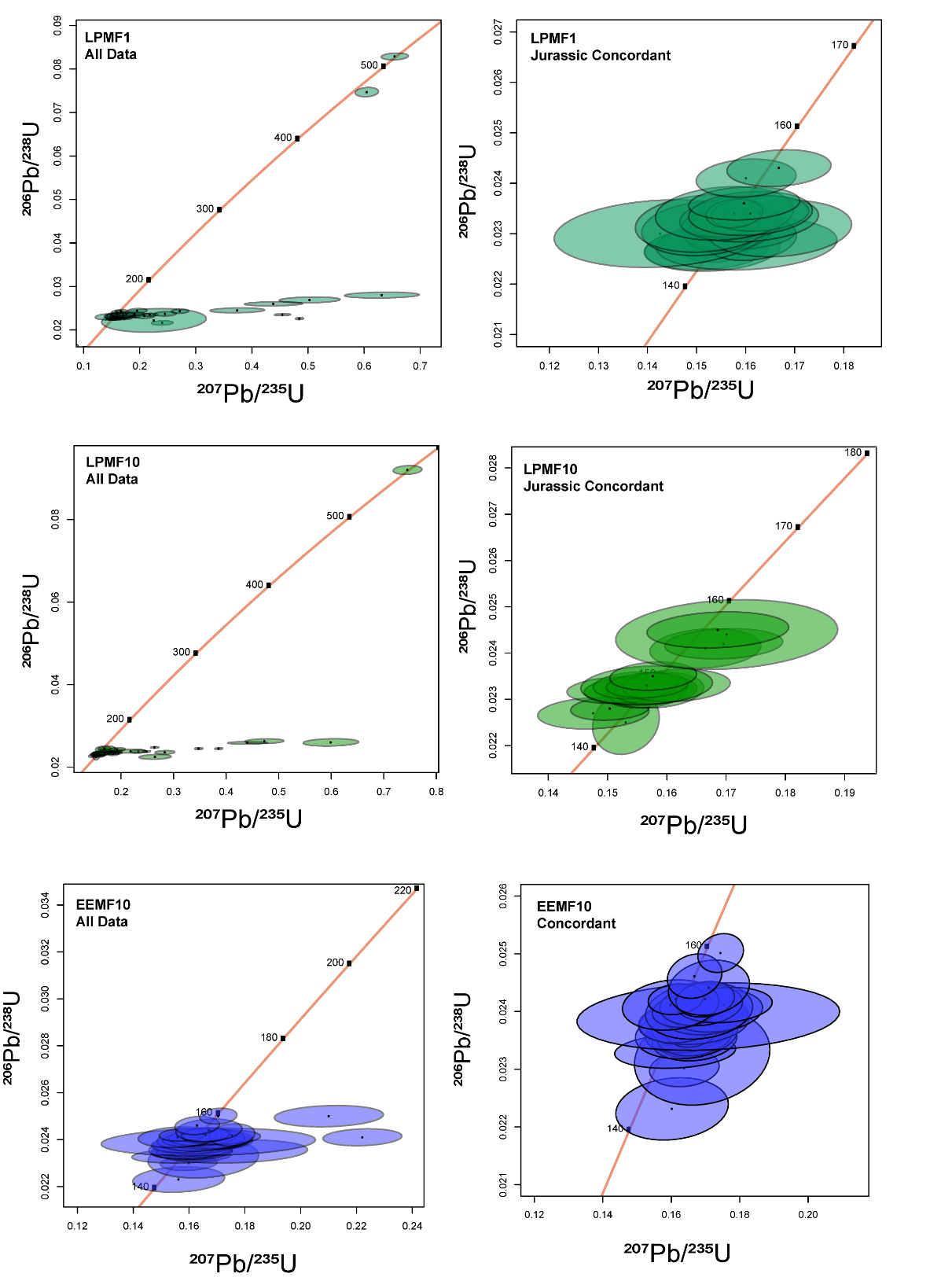


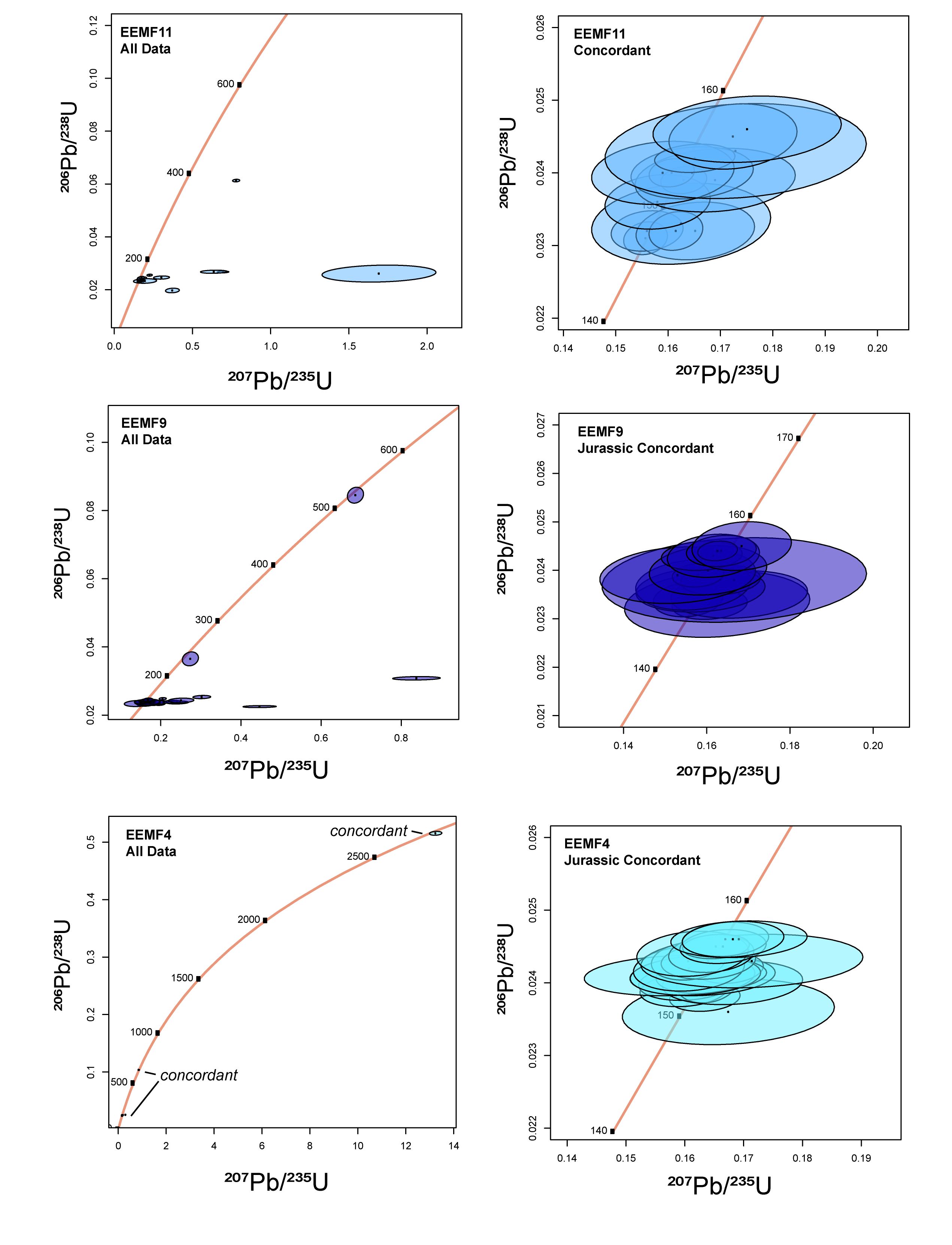


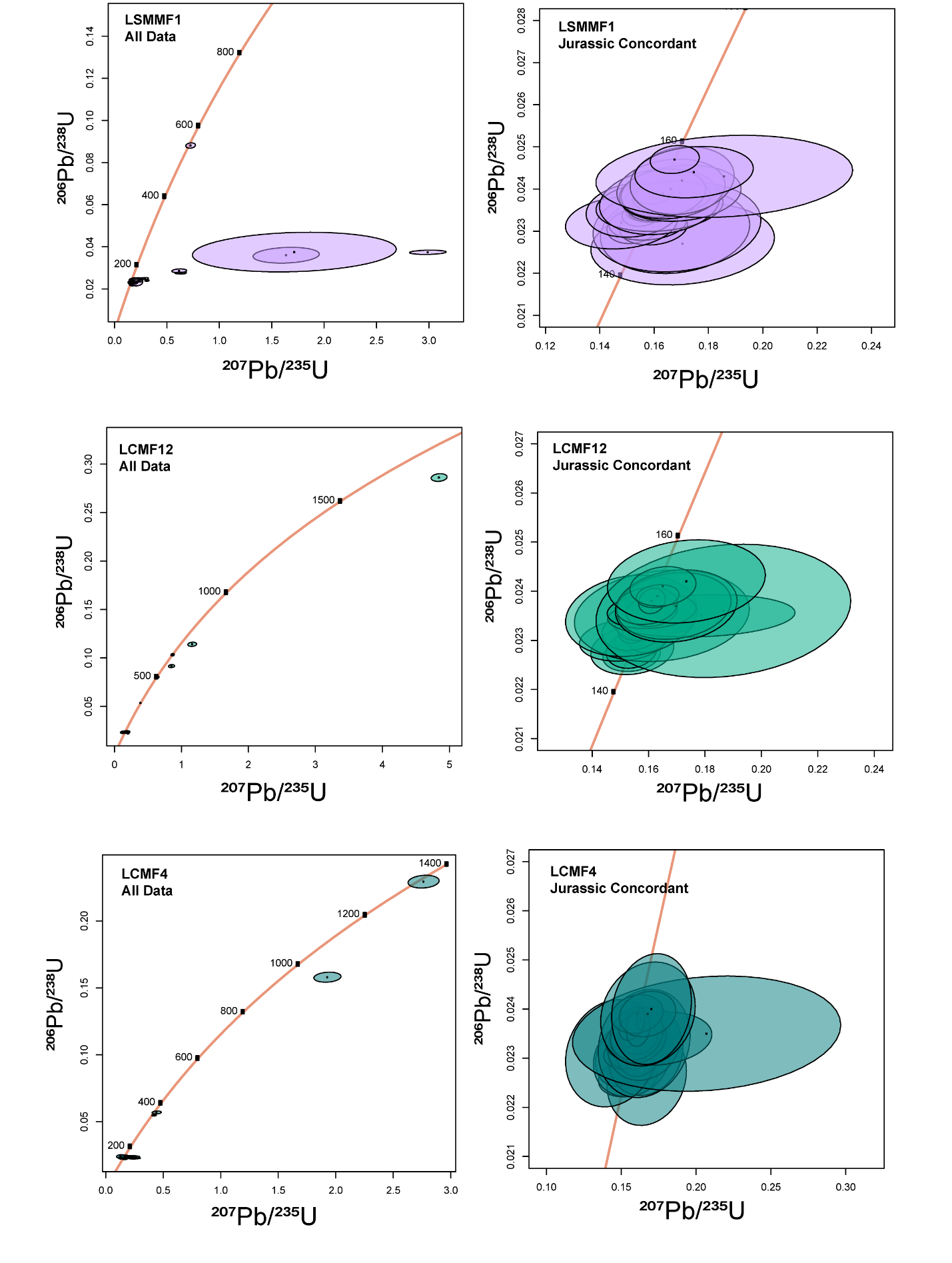


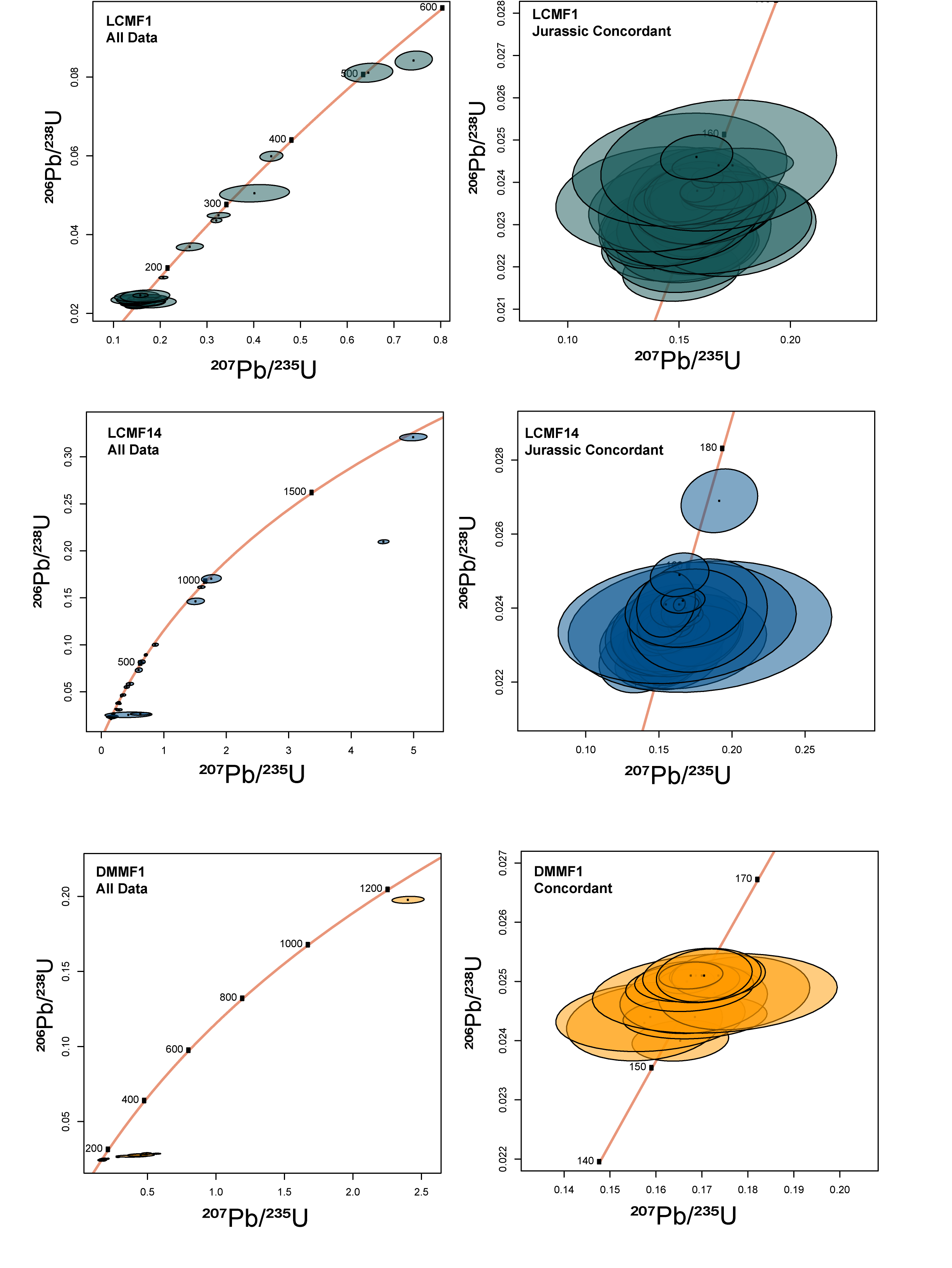

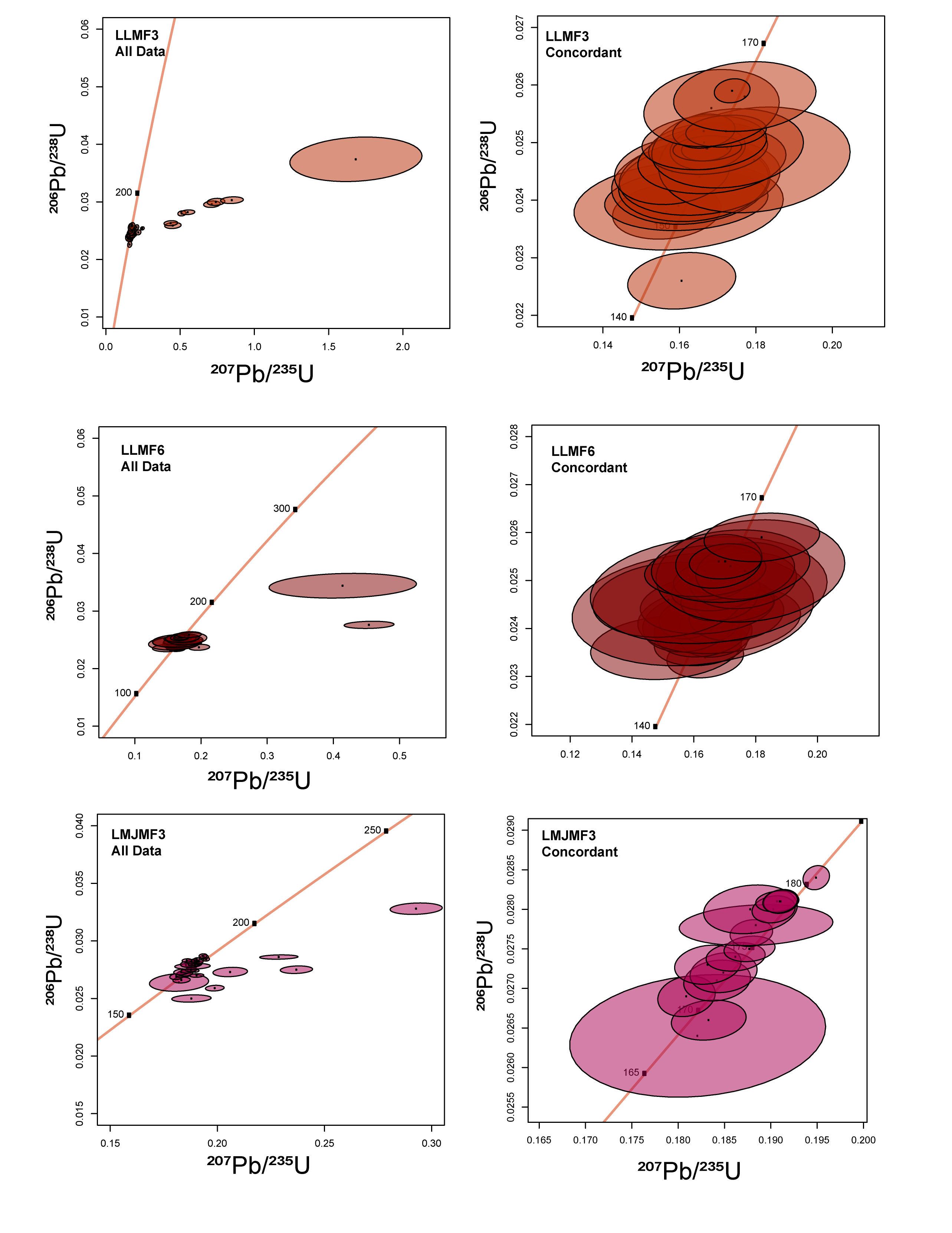


**SF3.** Mean weighted age diagrams demonstrating analyses used to calculated the average zircon crystallization age of each sample. Errors reported within each figure are those calculated for each sample from the original analyses; the final error calculated for error propagated from standards are reported in the text.


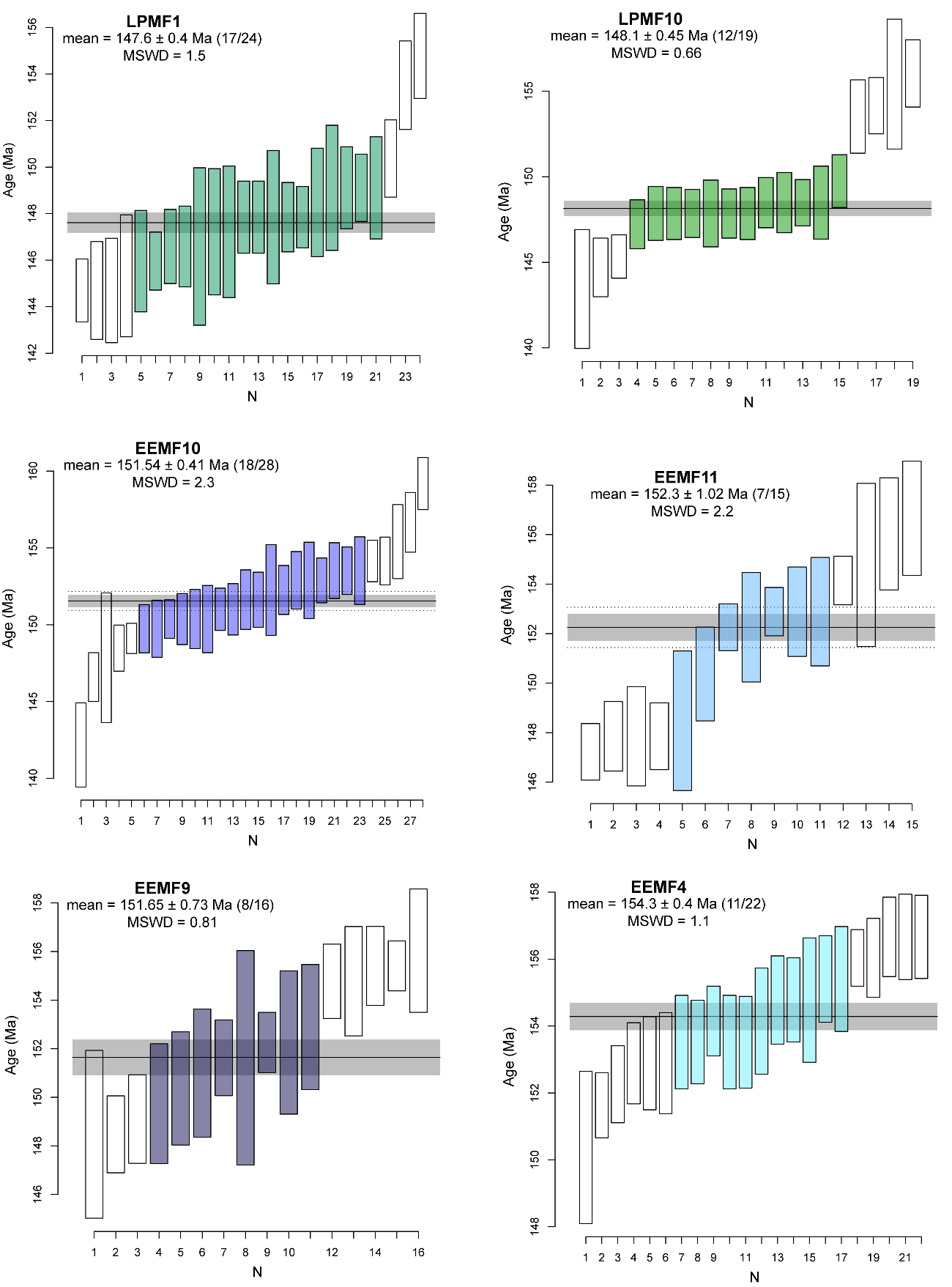


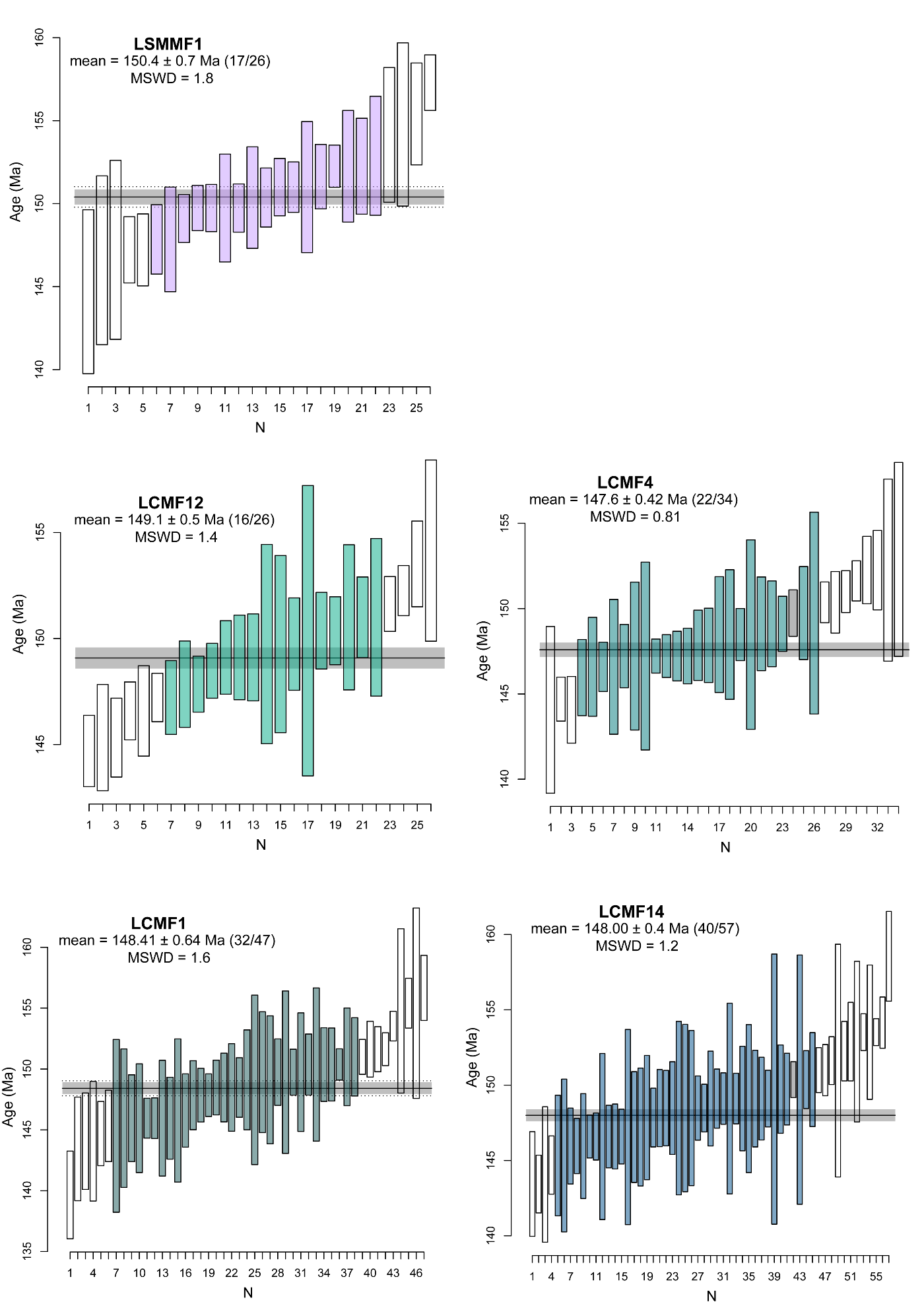


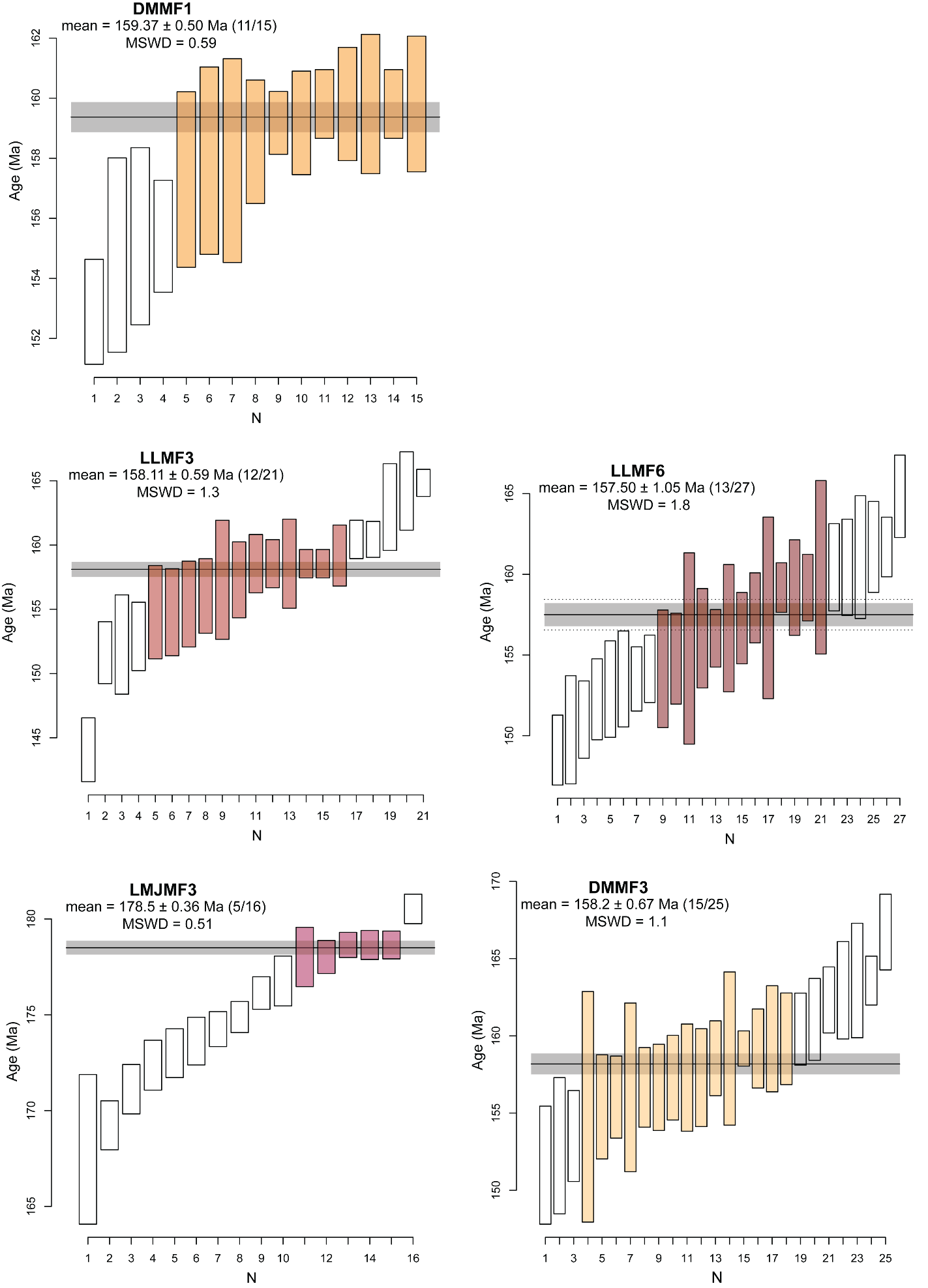


**SF4.** Continuation of Figure 10 in text, including LMJMF3. Zircon trace elements of (A) Hf (ppm) versus U/Yb, (B) Y (ppm) versus U/Yb, (C) Sm (ppm) versus Gd/Yb, and (D) Yb (ppm) versus U (ppm) are based on those designated by Grimes et al. (2015) to discriminate between different tectono-magmatic provenance.


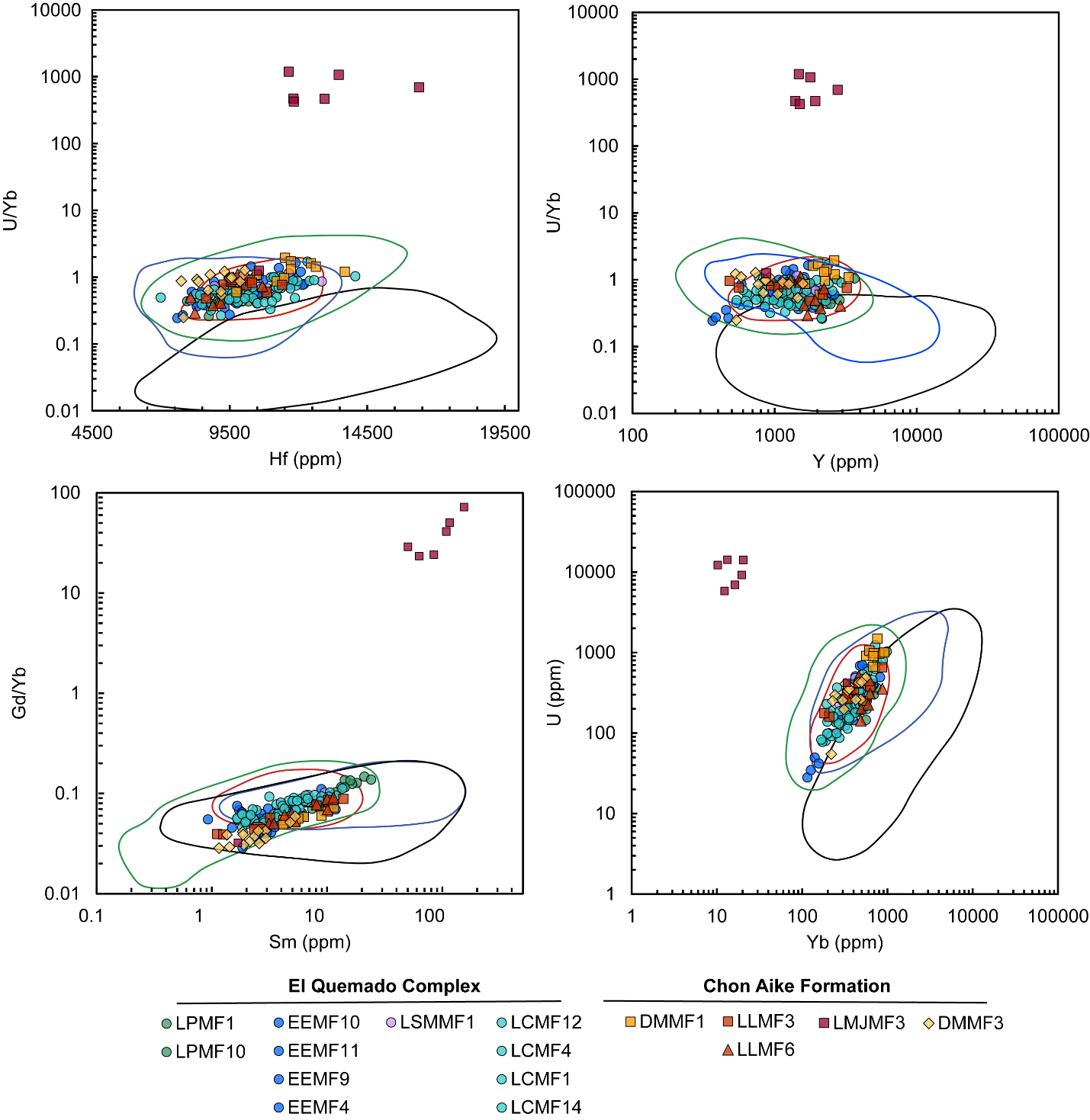

Supplement: Supplementary file 1 — Supplementary file1 (DOCX 4205 KB) [file 410_2023_2065_MOESM1_ESM.docx]
